# Doing More with Less: A Comparison of 16S Hypervariable Regions in Search of Defining the Shrimp Microbiota

**DOI:** 10.3390/microorganisms8010134

**Published:** 2020-01-17

**Authors:** Rodrigo García-López, Fernanda Cornejo-Granados, Alonso A. Lopez-Zavala, Filiberto Sánchez-López, Andrés Cota-Huízar, Rogerio R. Sotelo-Mundo, Abraham Guerrero, Alfredo Mendoza-Vargas, Bruno Gómez-Gil, Adrian Ochoa-Leyva

**Affiliations:** 1 Departamento de Microbiología Molecular, Instituto de Biotecnología (IBT), Universidad Nacional Autónoma de México (UNAM) Av. Universidad #2001, Col. Chamilpa, Cuernavaca, Morelos 62210, Mexico; rodrigo.garcia@mail.ibt.unam.mx (R.G.-L.); mafercg@ibt.unam.mx (F.C.-G.); fily@ibt.unam.mx (F.S.-L.); 2 Departamento de Ciencias Químico Biológicas, Universidad de Sonora (UNISON). Blvd., Rosales y Luis Encinas, Hermosillo, Sonora 83000, Mexico; alonsolopez.zavala@gmail.com; 3 Camarones el Renacimiento S.P.R. de R.I. Justino Rubio No. 26, Col. Ejidal, Higuera de Zaragoza, Sinaloa 81330, Mexico; el_andres_cota@hotmail.com; 4 Laboratorio de Estructura Biomolecular, Centro de Investigación en Alimentación y Desarrollo, A.C. Hermosillo, Sonora 83304, Mexico; rrs@ciad.mx; 5 Centro de Investigación en Alimentación y Desarrollo, A.C. Mazatlán, Sinaloa 82100, Mexico; aguerrero@ciad.mx (A.G.); bruno@ciad.mx (B.G.-G.); 6 Instituto Nacional de Medicina Genómica, Secretaría de Salud (INMEGEN), Periférico Sur No. 4809, Mexico 14610, Mexico; amendoza@inmegen.gob.mx

**Keywords:** *Litopenaeus vannamei* (*L. vannamei*), microbiota, bioinformatics, 16S rRNA, high-throughput sequencing, shrimp intestine, shrimp hepatopancreas, shrimp metagenomics

## Abstract

The shrimp has become the most valuable traded marine product in the world, and its microbiota plays an essential role in its development and overall health status. Massive high-throughput sequencing techniques using several hypervariable regions of the 16S rRNA gene are broadly applied in shrimp microbiota studies. However, it is essential to consider that the use of different hypervariable regions can influence the obtained data and the interpretation of the results. The present study compares the shrimp microbiota structure and composition obtained by three types of amplicons: one spanning both the V3 and V4 hypervariable regions (V3V4), one for the V3 region only (V3), and one for the V4 region only (V4) using the same experimental and bioinformatics protocols. Twenty-four samples from hepatopancreas and intestine were sequenced and evaluated using the GreenGenes and silva reference databases for clustering and taxonomic classification. In general, the V3V4 regions resulted in higher richness and diversity, followed by V3 and V4. All three regions establish an apparent clustering effect that discriminates between the two analyzed organs and describe a higher richness for the intestine and a higher diversity for the hepatopancreas samples. Proteobacteria was the most abundant phyla overall, and Cyanobacteria was more common in the intestine, whereas Firmicutes and Actinobacteria were more prevalent in hepatopancreas samples. Also, the genus *Vibrio* was significantly abundant in the intestine, as well as *Acinetobacter* and *Pseudomonas* in the hepatopancreas suggesting these taxa as markers for their respective organs independently of the sequenced region. The use of a single hypervariable region such as V3 may be a low-cost alternative that enables an adequate description of the shrimp microbiota, allowing for the development of strategies to continually monitor the microbial communities and detect changes that could indicate susceptibility to pathogens under real aquaculture conditions while the use of the full V3V4 regions can contribute to a more in-depth characterization of the microbial composition.

## 1. Introduction

In the last decade, shrimp species have emerged as one of the most economically relevant aquatic species worldwide, with a production of over 9.1 million tons (17% of the international market) in 2017, according to the Food and Agriculture Organization of the United Nations (FAO) [1]. As a result, shrimp farms have seen a surge in the demand of shrimp or prawn, including that of the Pacific whiteleg shrimp, *Litopenaeus vannamei*, currently the most cultured species, originally from the Western Pacific of Latin America [2]. Aquaculture shrimp production has far surpassed fishery production in terms of volume and economic share with over 5.5 million aquaculture tons (34.2 × 10^9^ USD) of the former compared to 3.6 M (14.7 × 10^9^ USD) of the latter [1].

The growing interest of *L. vannamei* as a culturable species has led to the study of the genetic lines, habitat and ecologic traits and, more recently, the publication of its genome and other meta-omic surveys have led to an integrative panorama of the species and its molecular context [3,4,5,6,7,8]. In this sense, research on the microbial communities associated with *L. vannamei* has shed light on the bacteria inhabiting its digestive tract (including the intestine and the hepatopancreas) under normal and different stress conditions [7,9]. As with many other organisms, the microbiota plays an essential role in the development and physiology of the shrimp, such as preventing the growth of pathogenic bacteria, modulating the immune response and nutrient absorption, regulating metabolic processes, and producing vitamins [10,11,12]. All these factors affect the overall growth of the organisms and are thus, relevant for their related economic activities [13]. 

To this date, most metagenomic studies focusing on the microbial communities depend on high-throughput sequencing of the 16S rRNA gene, making this analysis the standard profiling method for microbial taxonomy in shrimps [7,14,15,16]. Since read length limits most of the current high-throughput sequencing technologies, microbiota taxonomic surveys have regularly used amplicons that span one or two hypervariable regions within the 16S gene. Amplicons targeting multiple regions, such as the V1V2 (~330 bp) [17], V1V3 (~490 bp) [18], V4V5 (~390 bp) [19], and more commonly, the V3V4 regions (~460 bp) [7,12,13], have been used with varying results. Currently, these fragments are sequenced using MiSeq Illumina platforms, which produce paired-end (PE) reads that can be joined into ~600 nt [20] reads. The smaller independent V3 and V4 regions have also been successfully used for profiling the shrimp microbiota [16,21]. Due to their shorter length, the V3 or V4 region is suitable for small-length read technologies such as the HiSeq and the MiniSeq Illumina platforms, which uses PE reads that can be joined into ~300 nt [20]. The V4 region has been endorsed by the Earth Microbiome Project for large scale surveys of environmental microbiota [22].

The selection of various hypervariable regions used for 16S profiling produced different taxonomic resolutions, and these behave differently across the various ecological niches, while one region may be suitable for a specific environment, it might not be as accurate for a different one [23,24,25]. Technical variations among studies, such as protocols for DNA extraction, selected primers, PCR conditions, hypervariable regions, and sequencing platforms, show a significant impact on the shrimp microbiota structure [14]. Plus, factors such as cost and sequencing technologies, have limited the number of hypervariable regions used to characterize the differences of the microbiota. However, until now, there is no study using the same experimental and bioinformatics process on the same samples using several hypervariable regions to assess the limits in diversity and richness of each region without the intervention of technical biases. 

This work focuses on the comparison of three different types of amplicons spanning two hypervariable regions in the same set of samples to define the shrimp microbiota. We used the same DNA extraction protocol, PCR conditions, and bioinformatics protocols for the sequence analysis. Additionally, we compared two different 16S reference databases, GreenGenes and silva. Furthermore, we compared the differences in the taxonomic information retrieved by dual-region amplicons (V3V4) sequenced in an Illumina MiSeq platform with that captured by independent V3 and V4 amplicons sequenced in an Illumina MiniSeq platform. The results suggest that the more extended V3V4 region provides higher richness and diversity, and manages to capture a broader spectrum of the less prevalent species, whereas V3 and V4 retrieve a more similar composition between one another while being less expensive and providing enough variation to detect organ-specific differences at higher taxonomic levels. We found that the hypervariable region producing the highest diversity and richness was V3V4, followed by V3 and lastly V4 for the shrimp microbiota. Even though the V3V4 region may be the most informative region for future metagenomic studies, it is also more expensive to sequence than only V3. Thus we define that the V3 region may be a low-cost option that can be quickly sequenced and enables an adequate description of the hepatopancreas and intestine shrimp microbiota. Our results open the opportunity for the development of strategies to monitor the microbial communities constantly and to detect changes that could indicate susceptibility to shrimp pathogens or to analyze the effect of the shrimp diets in the microbial structure using a low and fast sequencing strategy.

## 2. Materials and Methods

### 2.1. Experimental Procedures

#### 2.1.1. Sample Collection

Four cultivated shrimps (average weight = 16.71 ± 2.23 g) were obtained from a farm of the Northwest Mexican Pacific area from Sinaloa (25°58′02.7″ N, 109°18′11.6″ W) and were identified as *Litopenaeus vannamei* by morphological keys [26]. The hepatopancreas and intestine from the same specimen were aseptically dissected in situ, kept in an RNA-later solution (Sigma-Aldrich, St. Louis, MO, USA), and stored at −80 °C until used. Thus, in total, we obtained DNA for four hepatopancreas and four intestines.

#### 2.1.2. DNA Extraction and Amplicon Preparation

Total DNA was extracted from all samples using the Quick-DNA Fecal/Soil Microbe Miniprep kit (Zymo research Cat. D6010, Irvine, CA, USA) following the manufacturer’s recommendations. The DNA concentration and integrity were assessed using agarose gel electrophoresis and Qubit (LifeTechnologies, Carlsbad, CA, USA), respectively. The 16S rRNA amplicons of the three-hypervariable regions were generated following Illumina manufacturer’s instructions. Primers 338F (5’-ACTCCTACGGGAGGCAGCAG-3′), and 533R (5’-TTACCGCGGCTGCTGGCAC-3′) were used to amplify the V3 region [27]. Primers 515F (5′- GTGCCAGCMGCCGCGGTAA-3′) and 806R (5′-GGACTACHVGGGTWTCTAAT-3′) were used for the V4 region [28]. Primers 341F (5’-CCTACGGGNGGCWGCAG-3′) and 805r (5’-GACTACHVGGGTATCTAATCC-3′) were used to amplify the V3V4 dual region [29]. The resulting amplicons were checked in agarose gel and purified using Ampure XP beads (Beckman Coulter, Inc., Brea, CA, USA). The purified amplicons were barcoded according to the Sequencing Library Preparation user’s guide (Illumina, San Diego, CA, USA). The concentration and size distribution of each library was assessed using the Qubit fluorometer and the Agilent 2100 Bioanalyzer (Agilent Technologies, Santa Clara, CA, USA). 

#### 2.1.3. Sequencing

Libraries for amplicon sequences targeted at the V3 and V4 16S rRNA hypervariable regions were prepared with MiniSeq Reagent kit (Illumina, San Diego, CA, USA) for 2 × 150 bp following the manufacturer’s protocol and sequenced in a paired-end (PE) format at the Research Center on Food and Development A. C. (CIAD) in Mazatlán, Sinaloa, Mexico. Libraries of V3V4 regions were prepared with MiSeq V2 Reagent kit (Illumina, San Diego, CA, USA) for 2 × 250 bp, following the manufacturer’s protocol and sequenced in a paired-end (PE) format at the National Institute of Genomic Medicine (INMEGEN) in Mexico City, Mexico.

### 2.2. Bioinformatic Methods

Figure S1 showed a diagram of the methods. 

#### 2.2.1. Quality Preprocessing

The amplification primers for the corresponding 16S regions and Illumina adaptors were removed with Cutadapt v2.0 [30]. A strict cleaning protocol with Prinseq v0.20.4 [31] was used to filter reads with low entropy including homopolymers and spurious repeats, trim low quality 3’ and 5’-ends based on Phred quality scores (q = 20 and 22, respectively) and remove sequences with low average quality (q = 22). Sequences shortened beyond overlap-producing lengths were removed based on the region and the associated type of sequencing (V3 < 120 bp, V4 < 150 bp, V3V4 < 170 bp). All clean PE sequences were subsequently joined with COPE v1.2.5 [32], and only joined sequences were considered for downstream analyses. A systematic random resampling without replacement approach was carried out with the seqtk suite [33] for each sample to cope with biases due to differential sequencing depth. Consequently, a total of 16,793 joined reads were drawn from each sample, reflecting the total abundance of the smallest sample in the whole set. Detailed information regarding data processing and analysis is provided as Supplementary Material.

#### 2.2.2. Clustering and Sequence Identification

Sequence clustering and taxonomic assignation were carried out separately for each 16S region using the QIIME2 v2019.1 suite [34]. Before clustering, each region set was dereplicated with Vsearch v2.7.0 [35] (sequences counts were kept in a contingency table for downstream abundance estimation). Clusters at 97% sequence identity based on the rRNA gene databases GreenGenes 13_5 [36] and the silva 132 [37] were used as the reference sequences for the present study. The V3V4 region fragments were extracted from the reference databases using the same primer sequences as the biological sets (341F and 805R) to train the scikit-learn machine learning classifier v0.19.1 [38] for taxonomic identification (hereafter, references and its derived products were referred as gg97 and silva97). 

An open-reference clustering approach was used to group dereplicated reads into operational taxonomic units (OTUs) using QIIME2 scripts. In this protocol, reference-based clusters were first identified as those aligned to the gg97 with 97% identity. Non-matching reads were clustered into de novo clusters at 97% identity. The procedure was repeated using silva97. Both sets of reference-based and de novo OTUs were referred to as BioSets. One BioSet was created for each reference database (gg and silva) and each hypervariable region (V3, V4, and V3V4). Singletons (i.e., OTUs appearing once in the table) for each region of BioSet_gg97 and BioSet_silva97 were removed. Reference and de novo chimeric OTUs were identified using Vsearch, and those sequences simultaneously identified by both methods were removed from the BioSets. Overlapping reference-based OTUs simultaneously captured by different regions were evaluated with Euler diagrams to assess unique item distribution among regions using the Euler library (v6.0.0) and in-house R scripts (v3.6.0) [39,40].

#### 2.2.3. Sparsity Reduction and Taxonomy

Data sparsity (i.e., a high ratio of zero to non-zero data produced by a wide data dispersion) was reduced with QIIME2 to obtain a representative core cluster set for all downstream analyses. To achieve this, only those OTUs accounting for more than 0.01% of the total item counts per region across all samples were kept. Rep-seq files corresponding to these OTUs were merged. The new overlapping ref-based clusters were analyzed with in-house R scripts.

Representative sequences from the core OTUs in the gg97 and silva97 BioSets were assigned a taxonomy with QIIME2s scikit-learn classifier using files created from the corresponding GreenGenes and silva databases (described above) as references. Euler diagrams were constructed in R for summarizing the overlap between the three regions at each taxonomic level. The total estimated richness (Chao1 extrapolated richness) and feature entropy (Shannon-Weaver’s index) per taxonomic level, plus OTUs, were calculated with vegan (v2.5-6) in R [41] to compare the gg97 and the silva97 BioSets.

#### 2.2.4. In Silico Sets of GreenGenes and Silva Databases for V3, V4 and V3V4 Regions

In order to explore the full potential of the reference database used for the diversity analyzes, two additional *in silico* sets were created by extracting the V3, V4, and V3V4 regions from the gg97 and the silva97 cluster sequences with QIIME’s feature-classifier extract-reads algorithm using the 338F and 533R primer for the V3 region, the 515F and 806R primers for the V4 region, and 341F and 805R for the V3V4 region. These sets captured any sequences available from the whole database that matched the corresponding regions. These sets captured any sequences available from the reference databases that matched the corresponding regions. The length distributions of the resulting *in silico* regions were compared with the BioSets.

#### 2.2.5. Recruitment Analyses

All diversity analyses were carried out using post-sparsity reduction (core) OTU tables from the gg97 BioSet. Multiple rarefactions at different sampling depths were drawn with vegan in R to evaluate the recruitment of the rarefied samples concerning the total unique features at a depth of the smallest sample and 10,000 items. The medians of each group at all depths were used to evaluate differences among the different regions and organ groups. Additionally, sample recruitment plots per region were created to evaluate the total number of samples required to capture the observed region core OTU diversity.

#### 2.2.6. Standardized Tables

A standardization method was applied to all taxonomy and OTU tables performing 10,000 Montecarlo repetitions (randomized, no-replacement, resampling iterations), unless stated otherwise, using the size of the smallest sample using vegan with in-house R scripts for the unbiased sample and group comparisons. The mean of the resulting 10,000 tables was used for each observation, and false discovery rate (FDR) correction was used on all statistics. Taxonomic and OTU composition and abundance were compared using the standardized tables. Paired statistical tests were calculated for all group permutations, using parametric t-tests whenever a Shapiro–Wilk test failed to reject normality (with α = 0.05) for both populations or non-parametric Wilcoxon signed-rank test in the remaining cases. Taxonomy differences per region and organ subgroup were compared by averaging the OTU observations of each of the groups and subjecting the resulting tables to total sum scaling (TSS relative abundance) calculated per subgroup. Also, to determine the maximum resolution of the taxonomic assignations per region and what percentage each region manages to capture out of the total informative labels per level, the total taxa recovered by each region and the percentage seen in each region were compared by removing all taxa without a valid label for the corresponding taxonomic level (i.e., empty, uncultured).

#### 2.2.7. Diversity Analyses

Within sample diversity (α-diversity) was evaluated with the Shannon and Chao1 indices, which were estimated for each permutation and averaged using vegan and in-house R scripts in the OTU table. The difference between observed and expected richness was calculated for each sample. Organ and region groups were compared for each iteration using non-parametric Wilcoxon signed-rank tests on all pairwise permutations, with FDR correction.

The degree of community differentiation (β-diversity) was evaluated with vegan using in-house R scripts to calculate Jaccard’s similarity coefficient and the Bray–Curtis index of (dis)similarity for each OTU and multilevel taxonomy tables for each of the 10,000 Montecarlo repetitions followed by a non-parametric adonis test (non-parametric multivariate analysis of variance; PERMANOVA) on organ and region group differences for each iteration. Statistical significance was FDR adjusted. Since de novo OTUs were region-specific, they were left out of this comparison, as they would artificially inflate between-region differences. Post-hoc testing was carried out for each iteration’s (dis)similarity matrices with pairwise adonis tests on all two-group permutations, adjusting with FDR. The standardized tables were subjected to multidimensional metric scaling (MDS) using a principal coordinate analysis (PCoA) ordination method with vegan and in-house R scripts. Additionally, UniFrac distances were calculated for each independent region from the gg97 BioSet OTUs by splitting their corresponding sequence collections for phylogenetic reconstruction with (SEPP) [42], followed by calculation of the distances with QIIME2 scripts. The OTU table was also split by region and standardized as described above and was the input for UniFrac distance calculations with QIIME scripts with resampling disabled. MDS and PERMANOVA tests were carried out using the same methodology. 

#### 2.2.8. Sample Correlation Analysis

Sample correlation matrices were constructed with the genus level taxonomic table from the summarized Montecarlo repetitions using in-house R scripts. Spearman’s rank correlations were calculated, and clustering was carried out with a weighted pair group method with averaging (WPGMA) approach.

#### 2.2.9. Differential Abundant Features

To identify taxonomic features that could explain most of the variation between groups, a linear discriminant analysis effect size (LEfSe) was calculated using the standardized species table [43]. Differential abundances were detected for each region, organ, and region-organ groupings with support between subgroups. The alpha cutoff was selected at 0.5 in both the Kruskal–Wallis (class) and the Wilcoxon tests (subclass). The linear discriminant analysis (LDA) effect cutoff was set to 1 (in log10 scale) for filtering relevant results from any group. Finally, tables were split to compare region and organs independently, allowing LDA eff = 2.

## 3. Results

### 3.1. Amplicon Sequencing of Biological Samples

Twenty-four amplicon samples from four shrimps (four intestines and four hepatopancreas) and three hypervariable regions were successfully sequenced. The eight V3 region samples produced 356,384 PE reads (mean = 44,548.00 ± 4797.64), the eight V4 region samples produced 439,636 PE reads (mean = 54,954.50 ± 23,579.83) and the eight V3V4 produced 406,036 PE reads (mean = 50,754.00 ± 22,147.79). Different filters were applied to each dataset to cope with differences derived from distinct sequencing platforms based on the specific Illumina quality specs. A table with the total number of reads in each of the quality processing steps is included in Table S1. Length filters (V3 < 120, V4 < 150, V3V4 < 170) removed 7.86% of V3 reads, 16.70% of V4 reads, and 4.92% of V3V4 reads. The 338F primer was removed from 99.8% of V3 R1 reads, the 533R primer was captured in 98.0% of the R2 reads, and artificial adapter constructs were removed in 1% of R1 and 0.2% of R2. In V4 reads, 515F was removed from 99.8% of R1 reads, 806R from 98.7% of R2 reads, and artificial constructs were detected in 0.8% R1 and 0.3% R2. In V3V4, the 341F primer was detected and removed in 99.9% of R1 reads, and in 100.0% of R2 reads, whereas artificial constructs were in 1.0% R1 and 0.2% R2. Flanking sequences in the resulting 5′ trimmed R1 and R2 confirmed a correct primer trimming. In subsequent steps, out of the resulting sets, 3.21% reads were lost to trimming and quality filters in the V3 set, whereas 2.71% and 5.98% of reads were removed from the V4 and V3V4 set, respectively, as trimming is not commonly expected to cause most sequences to be lost but rather be shortened. From these, the success of paired end joining was 88.59% for the V3 PE reads, 88.53% for V4, and 88.15% for V3V4 considering an expected overlap that was specifically adjusted to each region.

These resulted in a joined-read set of 912,694 complete sequences, including 279,931 joined sequences from V3 samples (mean = 34,991.38 ± 6478.94), 313,676 reads from V4 samples (mean = 39,209.50 ± 18,317.54) and 319,087 reads from V3V4 samples (mean = 39,885.88 ± 19,761.34). Considering the Phred quality scores per base position, the V3 region set showed the highest average (36.34), followed by the V4 (35.41) and V3V4 (34.77) regions. However, quality drops more markedly in the middle sections of the V3V4 datasets (Figure S2a). As expected, both MiniSeq derived sequencing sets (V3 and V4) presented a higher overall quality, but this is due to error probabilities are more accumulated in the larger paired sequences produced by MiSeq (Figure S2b). This is a typical effect of the sequencing-by-synthesis technology used by both platforms in which the last bases have minor quality. However, the lowest quality average for any position was similar among amplicons 33.81 in the V3, 30.71 in the V4, and 28.37. As expected, due to the insert size, sequencing-quality associated errors were more prevalent in the V3V4 but in a low proportion (Figure S2c).

**Figure 1:**
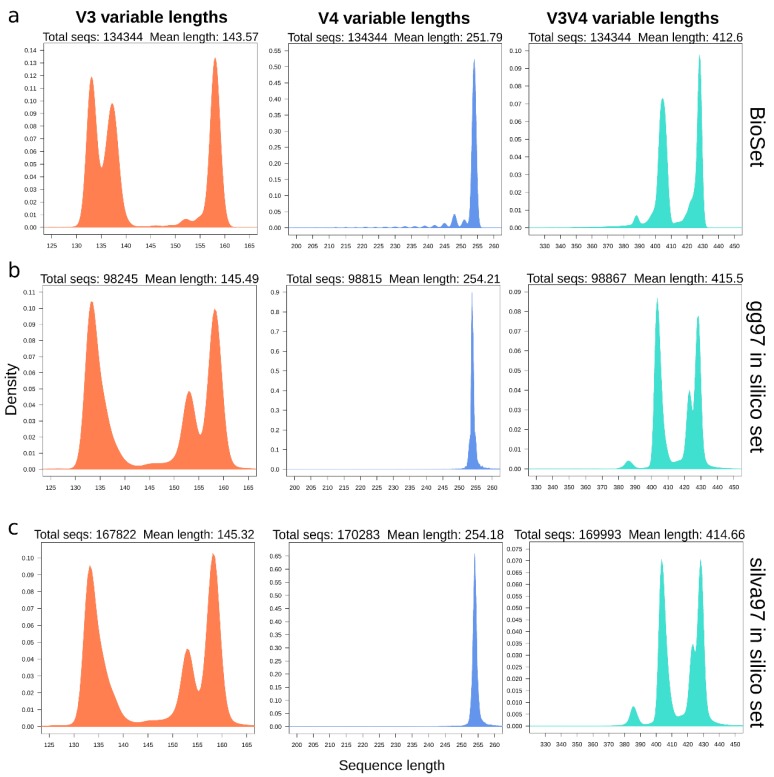
Sequence length distribution for V3 (left), V4 (center), V3V4 (right) experimental and *in silico* amplicons. (**a**) Experimental amplicons of Biological set. (**b**) Simulated amplicons constructed from the GreenGenes 97% identity clusters. (**c**) Simulated amplicons constructed from the silva 97% identity clusters.

Due that we obtained a different number of reads among samples after quality filters, we selected a randomized subsample of 16,793 joined sequences from each sample to standardize the sequencing depth for further analysis. This sequence depth normalization also allows each sample to contribute with the same number of sequences in the clustering formation, reducing the impact of clustering formation by sequence depth as new clusters formation depends on the number of input sequences. After that, we obtained a new subset with 403,032 sequences evenly distributed among the 24 samples (134,344 sequences per region). The length distribution of amplicons is shown in Figure 1a. This set was named amplicon BioSet. These sets constitute the input used for all downstream analyses and are available in NCBI’s SRA with Accession Numbers SAMN12913111-SAMN12913134 under BioProject PRJNA575880.

### 3.2. In Silico Amplicons Produce Length Distributions Similar to Experimental Ones

We extracted the *in silico* amplicons to compare with the experimental ones. There were a total of 99,322 and 172,222 sequences in the GreenGenes, and SILVA reference 97% identity OTUs. The corresponding experimental primers produced 98,245 (98.92%), 98,815 (99.49%), and 98,867 (99.54%) simulated amplicons from the V3, V4, and V3V4 regions, respectively, for GreenGenes (gg). Similarly, for silva (silva) we obtained 167,822 (97.45%), 170,283 (98.87%), and 169,993 (98.71%) simulated amplicons for V3, V4, and V3V4 regions, respectively. These amplicons represented the sequences theoretically retrieved from the reference database. The length distribution of *in silico* simulated amplicons is showed for gg in Figure 1b and silva in Figure 1c. As can be seen in Figure 1, the distribution of lengths spanning each of the three regions obtained from the biological amplicons (BioSet) is congruent to the simulated amplicons *in silico*. Although two peaks were found in the 130–140 region of the V3 from the BioSet, this area is well represented in the *in silico* sets flanked by the same length span, and differences may well be attributed to a larger and far more complete spectrum of the region lengths in the references.

### 3.3. Evaluation of OTU’s Similarity between Regions

**Figure 2:**
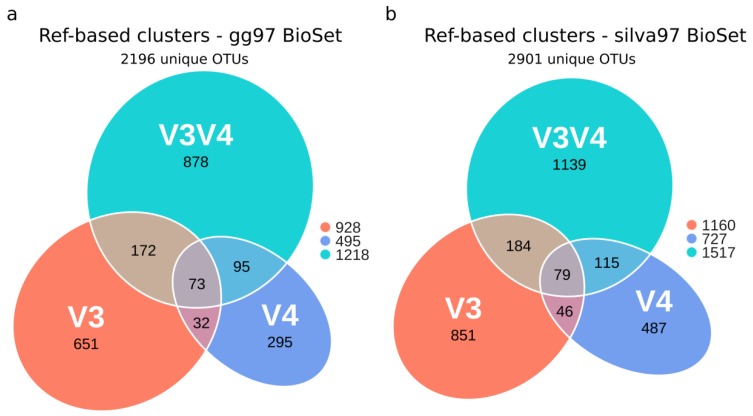
Euler diagram of unique operational taxonomic units (OTUs) captured by the V3, V4, and V3V4 regions in the gg97 and silva97 experimental BioSets. Only reference-based clusters were considered. (**a**) OTUs for the gg97 BioSet. (**b**) OTUs for the silva97 BioSet. The numbers of total unique clusters were indicated in the legend for each hypervariable region.

Open-reference clustering of the BioSet, followed by chimera filtering and taxonomic assignment grouped all input sequences into 928, 495, and 1218 different OTU clusters with 97% identity from the V3, V4, and V3V4 sets, respectively, using gg (Figure 2a, BioSet gg97). Similarly, 1160, 727, and 1517 clusters were produced with SILVA, respectively (Figure 2b, BioSet silva97). Of these, 2196 and 2901 OTUs were unique to gg and silva, respectively. As seen in Figure S3, most reads were classified into reference-based clusters, whereas a smaller percentage was grouped into de novo clusters. The sequences not assigned to a cluster were filtered as chimeras or removed due to low prevalence in the set. Only considering the reference-based sequences, most clusters were not detected in all three regions simultaneously for neither database (Figure 2). Further, the V3V4 region recruited a much larger number of unique clusters, followed by V3 and V4, regardless of the reference database.

### 3.4. GreenGenes and Silva Capture a Similar Number of Low-Level Taxonomies

**Figure 3:**
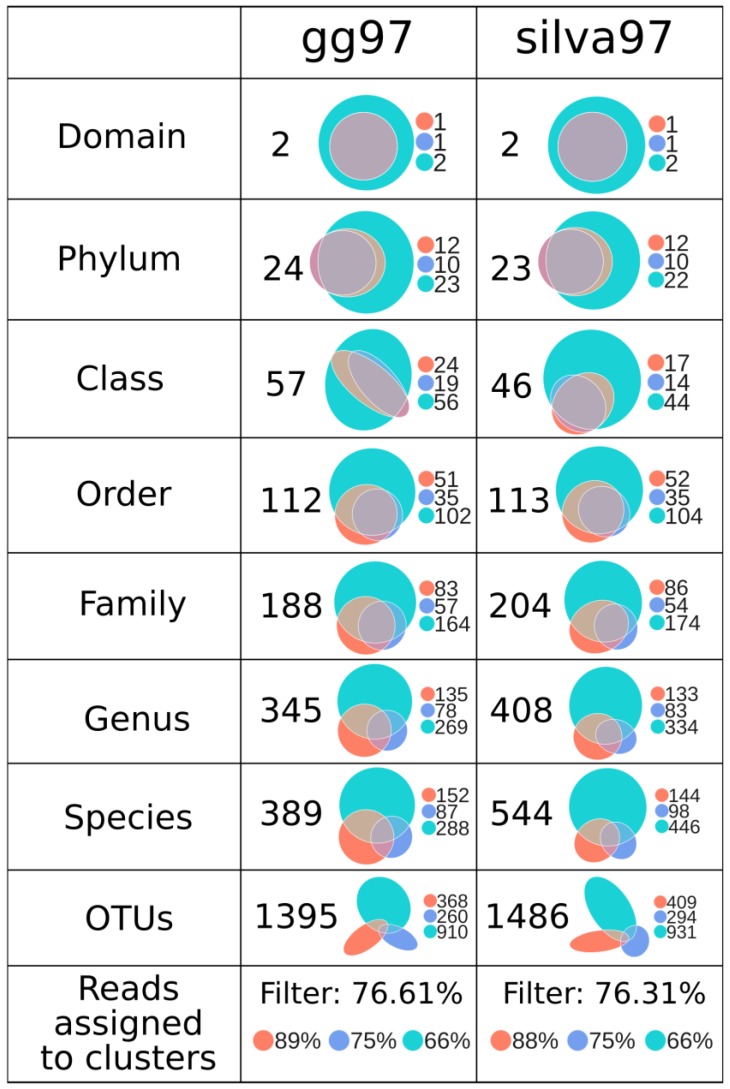
Taxa and OTU comparison from the gg97 and silva 97 BioSet clusters. Euler diagrams show the proportion of unique and shared OTUs for each region (V3: coral, V4: blue, V3V4: turquoise). Total unique taxa for all taxonomic levels plus raw OTUs (no taxonomic classification) were presented on the left part of each box. The legend on the right shows the total number of unique OTUs per region. Percentage of reads assigned to clusters and richness and diversity for each region are shown in the lower panel.

Before ecological analyses, a core microbiome set was determined by retaining only those OTUs accounting for more than 0.01% of the total observations per region. This threshold was selected to eliminate possible transitory bacteria and to eliminate the possible artificial sequence variability introduced by different sequencing platforms (Figure S2), resulting in new artifacts clusters but with low sequence abundance. The number of total unique OTUs per sample was highly similar between databases at the same region, showing near-perfect sample correlation for the complete sample set (Spearman’s rho = 0.997), as well as for all regions (V3 = 0.976, V4 = 0.952, V3V4 = 1) (Figure S4). Figure 3 shows the taxonomy comparison of the OTUs assigned by GreenGenes and silva. As seen in the upper panels, both the gg97 and silva97 capture a similar number of unique domains, phyla and orders, which were similarly distributed across the three regions. The higher numbers of unique taxonomies at different taxonomical levels were obtained for V3V4 followed by V3 and finally V4. The numbers of unique taxonomies at phylum, class, order, and family levels were very similar among gg and silva for all three regions. Contrary, there were more genus, species, and OTUs in the silva97 sets; however, further exploration revealed that several of these derived on uninformative labels such as “uncultured”, “bacterium”, and technical clade labels. After these analyses, we selected the gg97 BioSet for all downstream ecological analyses. It is important to note that from a total of 7400 unique OTUs for the three regions obtained in the gg97, 192 did not have classification beyond domain Bacteria, accounting for 2.59% of the total sequences. However, after frequency filters, only seven unclassified OTUs remained in the final table used for ecological analysis, but these only accounts for a total of 168 sequences (0.46% of the total observations). This suggests a non-significant impact of the unclassified OTUs in our abundance taxonomic analysis.

Interestingly, considering all the taxonomy levels (Figure 3, panel gg97), 13.70%, 20.23%, and 66.07% of the taxa simultaneously appeared in three, two, and one region, respectively. This indicates that only the 13.70% of the total taxa can be found using any of the three amplicon sequencing strategies. However, the taxa appeared in the three regions accounted for 86.76% of the total sequences, while only 7.23% appeared in two regions and 6.01% in one region. This suggests that few taxa account for the majority of sequences that can be captured by three regions. The rest of the taxa are individualities of each region. Furthermore, when we analyzed at each taxonomic level, the taxa shared in all three regions was for phylum = 37.50%, class = 28.07%, order = 25.89%, family = 20.21%, genus = 9.28%, species = 7.20%. Although, these particular taxa accounted for the majority of the sequences (phylum = 98.41%, class = 97.03%, order = 95.28%, family = 89.07%, genus = 69.43%, species = 58.75%).

### 3.5. Recruitment Analyses Showed a Higher Number of Unique OTUs in V3V4 Followed by V3 and V4

**Figure 4:**
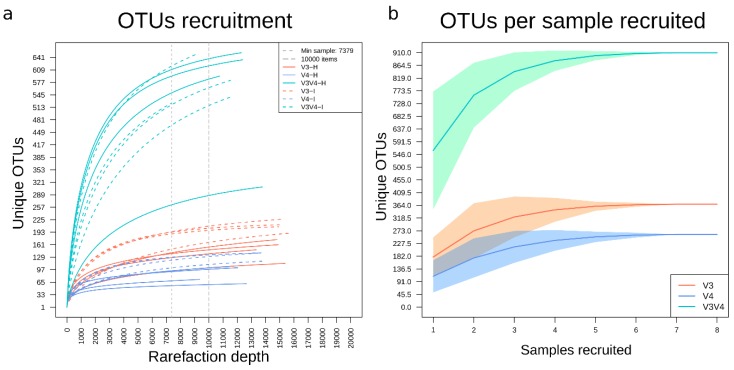
OTU recruitment at different rarefaction depths and number of samples. (**a**) Recruitment plot depicting the total number of unique OTUs captured by each sample in the gg97 BioSet. Each colored line represents a sample type (organ and region). H stands for hepatopancreas, I for intestine (intestine). The size of the smallest sample is marked with vertical dashed lines, as well as the 10,000 reads mark. (**b**) OTUs samples recruitment per region. The mean and density were calculated over OTUs 10,000 iterations.

The recruitment plot from the rarefied OTUs (Figure 4a) showed that the V3 and V4 samples had effectively captured most of the expected variation with V3 showing a more significant number of unique OTUs. Most curves for V3V4 samples, however, showed steeper slopes at higher rarefaction depth and did not seem to reach a plateau, most notably in the intestine samples when compared to their hepatopancreas counterparts. This data suggests that a higher sequencing depth of V3V4 samples may be necessary to reach the plateau. As a complementary analysis, random sample recruitment showed that a minimum of six samples was required to recover the OTU variability reported per each region in this study (Figure 4b). Although the V3V4 region is the most variable set, it takes only four samples to establish that V3 has a higher OTU variability than V4.

### 3.6. The Taxonomic Composition is Region and Organ Dependent

**Figure 5:**
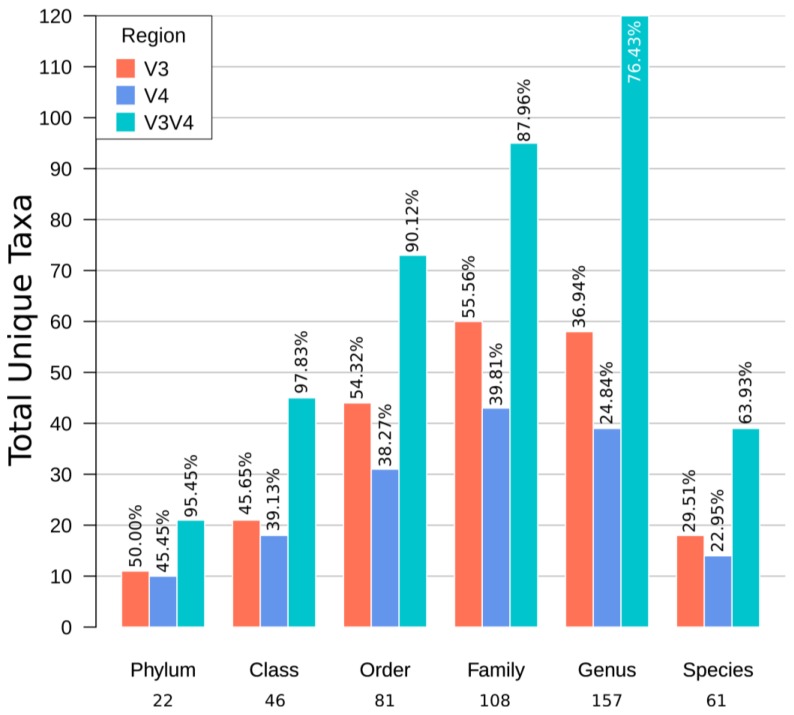
Total taxa and a percentage per taxonomic level and region. The total taxa at the phylum, class, order, family, genus, and species levels are shown in the *x*-axis. The height of the bars represent the total taxa found per region, along with the percentage. Only informative taxa labels were considered.

Figure 5 summarizes the total number of taxa with scientific taxonomic names to compare the performance of the taxonomical classification of each region at any given level. Thus, only taxa with scientific taxonomic names at the last level of classification were considered for this comparison. We found that each region captures a different percentage of the total unique observed taxa with scientific names. In all taxonomic levels, the same pattern is observed, with the V3V4 region capturing approximately twice the number of unique taxa than any of their single region sets. Interestingly, V3 also shows a higher number of unique taxa, despite having a shorter length than its V4 counterpart. Genus was the taxonomic level with most tags, indicating that the genus level may be the optimal for taxonomic comparisons with this methodology, most notably with the V3V4 region, which captured 76.43% of all obtained genera, followed by V3 and V4. In contrast, single region sets, showed a slightly higher number of families (V3 = 55.56%, V4 = 39.81%) than genera (V3 = 36.94%, V4 = 24.84%), suggesting their ideal resolution may lie at that level.

**Figure 6:**
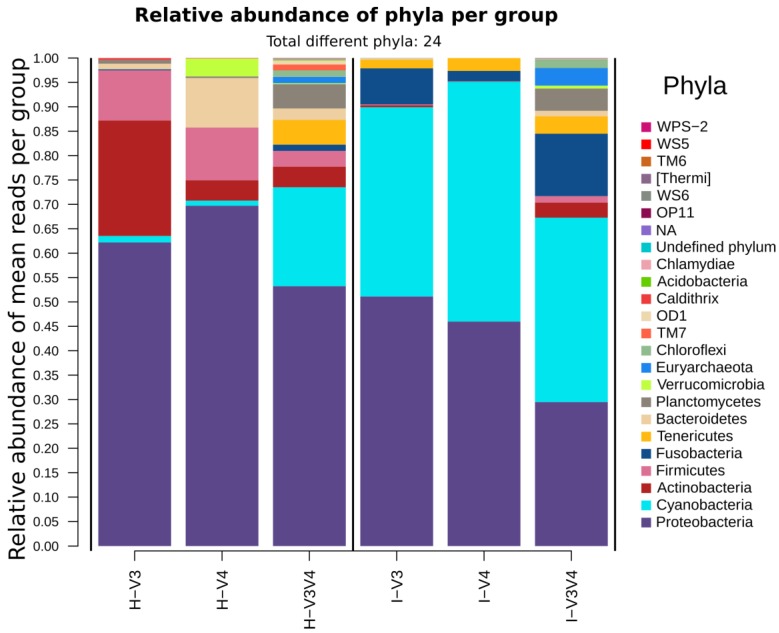
Relative abundances at the phylum level per region/organ groups. Phyla are ordered by abundance. Groups on the left show hepatopancreas samples (H), while groups on the right show intestine samples (I).

Considering the whole taxonomic composition, Figure 6 shows that the averages of total phyla observations (total sum scaling relative abundance calculated over the mean per subgroup of every observation) were more different among organs than regions. The V3V4 was the most diverse region, irrespective of the organ, as recovered 11 out of 22 informative phyla, plus, this was the only region to recover any taxa from the Archaea domain. On the other hand, V3 and V4 presented a more similar taxonomic composition and showed that marine Caldithrix as an exclusive phylum to these regions. Notably, all regions assessed Proteobacteria as the most abundant phyla, accounting for 51.96 ± 21.13% of the whole set, with a higher prevalence in the hepatopancreas (61.72 ± 18.09%) than in intestine (42.20 ± 19.95%). Contrastingly, the second most abundant phyla was clearly Cyanobacteria for the intestine (41.92 ± 11.71%) but was not so consistent in the hepatopancreas, where the V3V4 region estimates Cyanobacteria with 7.55 ± 11.71% while V3 detects Actinobacteria as the second most abundant with 23.68 ± 3.88% and V4 Firmicutes with 10.27 ± 17.76%.

The remaining phyla were more unevenly distributed among samples, often detected preferentially in particular groups such as Actinobacteria, with a more substantial prevalence in the H-V3 group (mean = 23.68 ± 3.88%), and Bacteroidetes, in the H-V3 group (mean = 10.13 ± 1.90%). Some taxa were preferentially detected in particular groups but with a high within-group variation such as Fusobacteria, in groups I-V3 (7.38 ± 10.30%) and V3I-V4 (12.79 ± 12.94%) or Firmicutes in H-V3 (mean = 10.27 ± 17.76%) and H-V4 (mean = 10.81 ± 16.86%).

**Figure 7:**
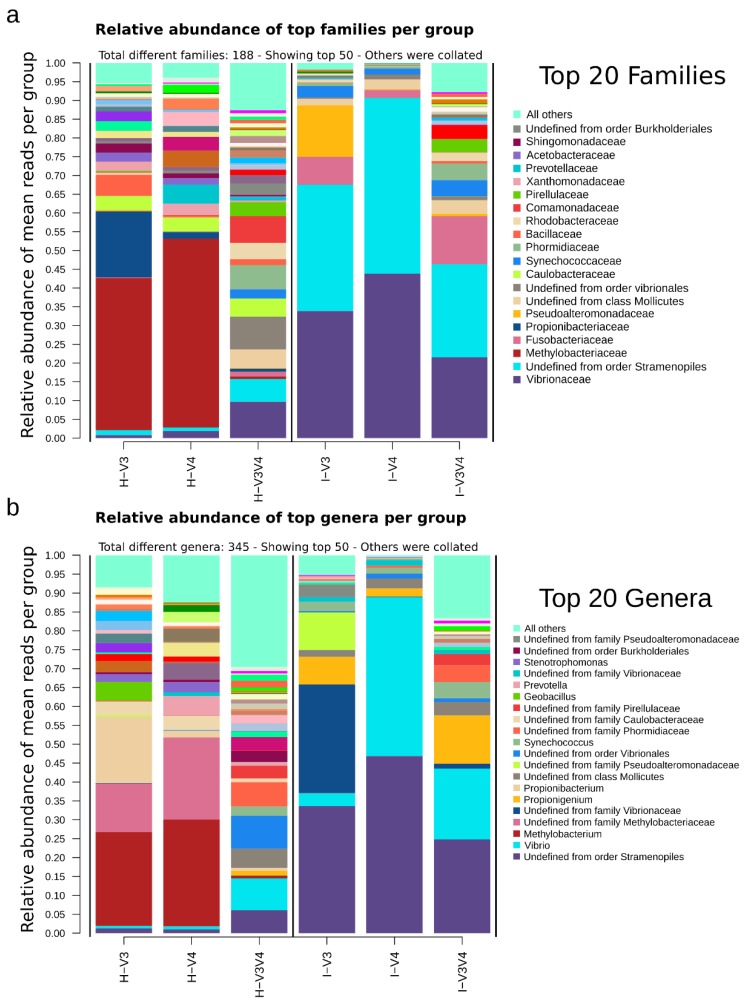
Relative abundance at Family and genus per region-organ groups. Taxa are ordered by abundance. Only the top 20 were shown, and the sum of the rest was shown as all others. (**a**) Family and (**b**) genus.

Differences between regions were more evident in higher taxonomic levels, as seen for the top 50 families shown in Figure 7 (per group) and Figure S5 (per sample). Families also showed a greater diversity in the V3V4 region, regardless of the organ. Of the total families, 90 were only detected in V3V4 samples, whereas V3 exclusives were 16, and V4 exclusives were 4. Among the most abundant families, Vibrionaceae was preferentially detected in intestine samples (33.08 ± 18.97%) when compared to their hepatopancreas counterpart (4.12 ± 8.50%)(*p*-value = 0.001).

A similar case was an unidentified family from what appears to be a Stramenopiles’ chloroplast (but classified as Cyanobacteria) with mean = 18.95 ± 22.25%. These were preferentially recovered by samples from intestine samples (mean = 35.1 ± 21.24%) when compared to those from hepatopancreas (mean = 2.79 ± 3.78%) (*p*-value < 0.001). Other examples from less abundant families with a larger intra-group variability include Fusobacteriaceae, which was more prevalent in the intestine samples (7.43 ± 9.93%) when compared to the hepatopancreas (0.5 ± 1.42%), and Caulobacteraceae, which was more prevalent in the hepatopancreas (4.11 ± 3.05) while mostly absent from the intestine samples (0.02 ± 0.03). Some other families were unevenly distributed but preferentially recovered in some groups, such as family Methylobacteriaceae, prominently found in H-V3 (40.55 ± 20.28%) and H-V4 (50.27 ± 20.88%), or the Propionibacteriaceae, prevalent in the H-V3 group (17.57 ± 6.21%), and the Pseudoalteromonadaceae in I-V3 (13.72 ± 24.55%). Also worth noting, genera within the top 50, accounted for >10% in most groups, which reflects a higher variability, especially seen in the V3V4 datasets (these account for >30% and >15% in the hepatopancreas and intestine samples, respectively). Additionally, most Vibrionaceae reported in the family level, were from genus *Vibrio*, identified mostly in the I-V4 and I-V3V4. Also, most identified Methylobacteraceae were from the *Methylobacterium* genus, and most Fusobacteriacea were from the genus *Propionibacterium*.

### 3.7. V3V4 Showed the More Considerable Richness and Diversity, Followed by V3 and V4

**Figure 8:**
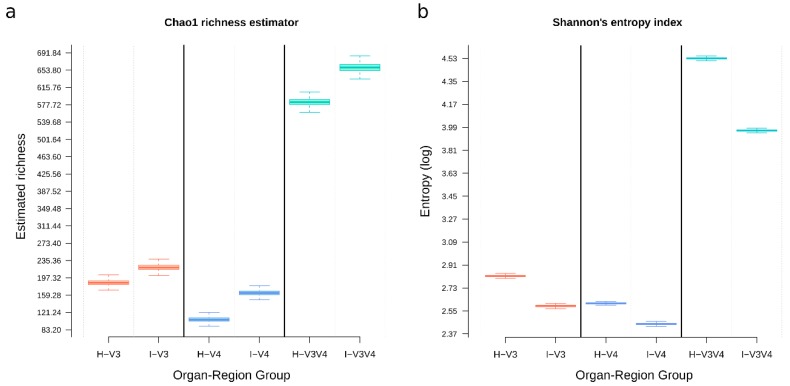
OTU richness (Chao1) and diversity (Shannon) by organ-region set. The distribution of means from index values in 10,000 Montecarlo repetitions drawn from the original table. Outliers are not shown. (**a**) Chao 1 estimated richness and (**b**) Shannon–Weaver’s entropy.

Overall, the V3V4 region showed the most significant number of expected OTUs, with a mean Chao1 estimate of 613.97 ± 112.72, followed by the V3 region, with 188.97 ± 32.67 and V4 with 119.23 ± 32.67 (Figure 8). Similarly, the Shannon index demonstrated a more considerable OTU diversity in the V3V4 region with 4.30 ± 0.85, while the single regions showed more similar values (V3 = 2.52 ± 0.43; V4 = 2.44 ± 0.25). The expected theoretical richness was compared to the observed richness and showed that an additional 17.58 ± 5.85% OTUs might potentially be recovered per sample on average. Max richness differences (observed vs. estimated) were more pronounced in the V3 region (19.39 ± 6.43%) than those reported for V4 (17.41 ± 5.48%) and V3V4 samples (15.95 ± 5.03%), suggesting more additional unique OTUs may be recoverable from the V3 region. Intestines have more richness than hepatopancreas independently of the sequenced region (Figure 8a). Interestingly, the Shannon index showed the opposite tendency (Figure 8b), with all hepatopancreas groups showing a higher diversity than the intestine. The V4 diversity was, in general, less diverse than the V3 region, but both were far less diverse than the V3V4. However, only the diversity differences between the V3V4 vs. either the V3 or the V4 regions with different organs were statistically significant (Wilcoxon signed-rank tests *q*-values: H-V3 vs. H-V3V4 = 0.114; H-V3 vs. I-V3V4 = 0.029; H-V4_vs_H-V3V4 = 0.057; H-V4_vs_I-V3V4 = 0.029).

### 3.8. Beta Diversity Shows that Most Region-Organ Groups are Different in Terms of Composition and Abundance

**Figure 9:**
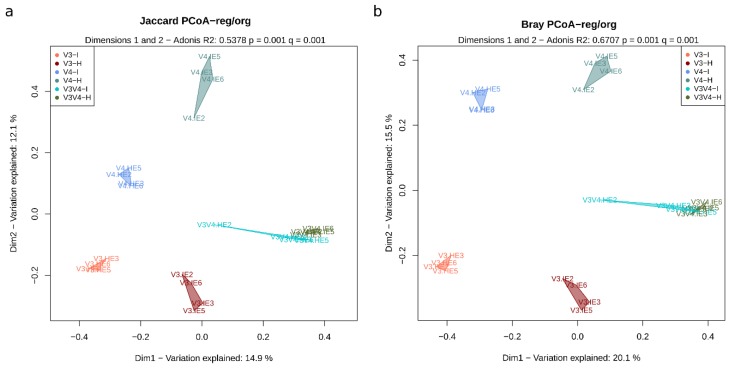
Multidimensional scaling of differences in OTU composition and abundance. Polygons highlight each cluster formed by region-organ sets. (**a**) The first two linear combinations of the PCoA constructed from the Jaccard similarity index (composition) matrix, comparing all samples. (**b**) First two dimensions of the principal coordinate analysis (PCoA) from the Bray–Curtis (abundance) dissimilarity index comparing all samples. Adonis *p* values were calculated between groups and false discovery rate (FDR)-adjusted for the 10,000 Montecarlo iterations.

Ecological differences between the samples were evaluated in terms of abundance (Bray–Curtis dissimilarities) and composition (presence/absence; Jaccard similarities) over 10,000 Montecarlo permutations of the core OTU table. The principal coordinate analysis (PCoA) multidimensional scaling (MDS) ordination for the first two dimensions is shown in Figure 9 for the different OTUs. All group differences shown between region-organ groups (H-V3, I-V3, H-V4, I-V4, H-V3V4, I-V3V4) were statistically significant (*q* values < 0.05 over pairwise *post-hoc* adonis tests), except for the V3V4 intestine and V3V4 hepatopancreas, which clustered together in a single V3V4 region cluster, as seen in both panels of the Figure 9. The differences between organs were most evident within both the V3 and V4 regions but not within the V3V4 set, both in terms of composition (Figure 9a) and abundance (Figure 9b). The organs can be differentiated with smaller regions (V3 and V4), while V3V4 makes both organ groups appear more homogeneous when all samples are compared. Nonetheless, when we observe only V3V4, V3, or V4 regions in the PCoA, the organs show a clear distinction (Figure S6).

According to Adonis tests, a more significant percentage of variation was explained by the abundance differences between region-organ (6 groups) (Figure 9b, R^2^ = 0.670) than by composition (Figure 9a, R^2^ = 0.537). Three main groups of samples were detected in the first dimension; the first group was formed by V3-H and V4-H, the second by V3-I and V4-I, and a third one containing the rest of the V3V4 samples (Figure 9b). The second dimension effectively clustered samples by region (V3, V4, and V3V4). When we performed the adonis tests between organ groups (all hepatopancreas samples vs. all intestine), the composition similarity matrices (Jaccard) showed a low R^2^ of 0.107 and 0.134 for organ and sequencing platform, respectively, while grouping by region (all V3, all V4, all V3V4) had an R^2^ of 0.250, demonstrating the inner variation within groups masked variation within both groupings. This data also suggests that considering all samples, the region explained more variability than the organ. All these differences were statistically significant (*q* < 0.05). However, the statistical comparisons of all region-organ (6 groups) showed that the differences between samples from different regions (R^2^ = 0.379 ± 0.047) explained less variation than organ differences within samples in each region (hepatopancreas vs. intestine samples within the same region; R^2^ = 0.418 ± 0.032), meaning region clusters were consistent with those detected in the PCoA (Figure 9) and could successfully differentiate samples by organ. 

In the abundance matrices (Bray–Curtis), most differences between groups were, in general, reported to be larger than in composition matrices. The organs across the whole set had a higher within-group dissimilarity (R^2^ of 0.131 in PERMANOVA tests), followed by the sequencing platform (R^2^ of 0.172), regions (R^2^ of 0.324), and then by region/organ groupings (R^2^ of 0.671) meaning that clusters were better defined when samples were separated by both organ and region. In all cases, *post-hoc* testing demonstrated that differences between groups were also statistically significant (*q* < 0.05). As with the composition, higher similarities were observed between regions (R^2^ = 0.497 ± 0.088) than within groups from the same region (R^2^ = 0.559 ± 0.045), meaning the organs most likely contributed to the overall differences within each region group.

Since OTUs were associated with entirely different representative sequences, there was no way to integrate phylogenetic distances to the comparison of β-diversity using the whole three regions. Instead, phylogenetic information was built for each region separately using UniFrac distances (Figure S6). Ordinations of UniFrac unweighted (composition) and weighted (abundance) matrices revealed that samples were successfully grouped by organ, independently of the region. However, among the V3V4 samples, a lower R^2^ was observed (0.391) as compared to V3 (0.708) and V4 (0.665), implying that even when phylogenetic information was added, organ clusters in V3V4 showed the lowest discriminatory power. The V3 region obtained the highest discriminatory power among organs.

### 3.9. Correlation Analyses Show that Sample Similarity is Heavily Influenced by Organ in the V3 and V4

**Figure 10:**
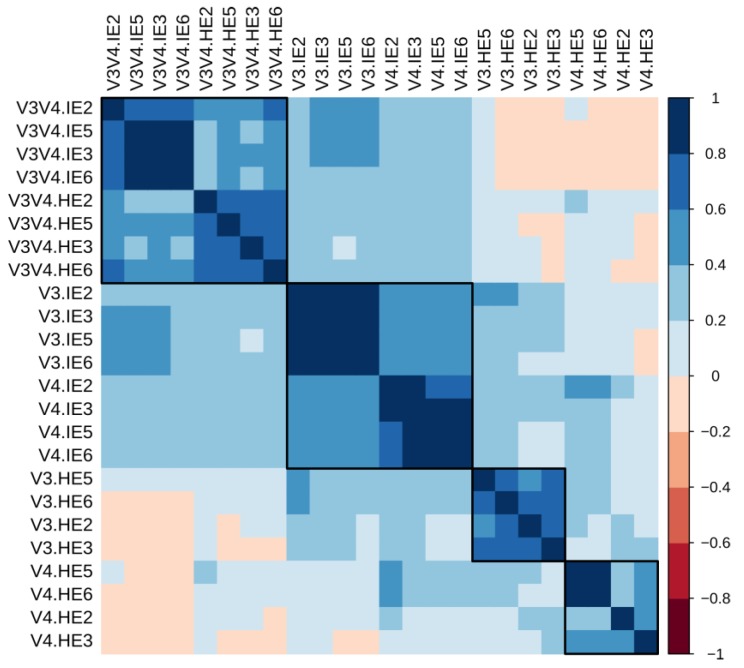
Correlation heatmap comparing the distribution of genera among all samples. Heatmap shows WPGMA hierarchical clustering of the samples according to their genera distribution similarities. The scale of variation was set to a discrete scale rather than a gradient for a more straightforward interpretation. Clustering showed as black square linings support the existence of a series of samples with genera distribution.

In the species taxonomy table, only 61 taxa had an informative species-level label. In consequence, the genus-level table containing 157 informative labels was selected for evaluating sample similarity using Spearman’s correlations. Figure 10 shows the matrix of correlations between samples, presenting WPGMA-derived clusters formed by highly correlated samples. In single regions (V3 and V4), samples from the same organ showed a more similar genera composition between one another, regardless of the region. Contrastingly, V3V4 samples were more similar between each other, forming a region-based cluster, and they were negatively correlated to most hepatopancreas samples in the V3 and V4 samples. Also, there was a greater congruence between the V3-I, and the V4-I subsets, shown in a single cluster, and then between them and the V3V4 cluster, meaning the intestine samples in the smaller regions are the ones that best resemble the abundances in the larger region. Notably, the highest correlation between samples (R^2^ < 0.89) was observed between different samples from the same regions. Cross-region correlations were, in general, weaker, all of them with an R^2^ < 0.53. These two observations show that genera distribution is more congruent to other samples from the same region than between different regions, which may be due to a tradeoff of differential genera dominance per region and the composition of trailing minority species.

### 3.10. Differential Abundance Analyses Detect More Features Specific to Organs than to Regions

**Figure 11:**
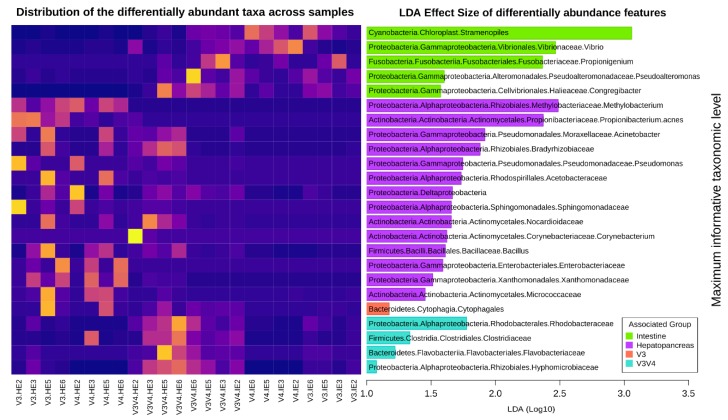
Distribution of differentially abundant multi-level taxa selected by the Linear discriminant analys Effect Size (LEfSe). The heatmap represents the scaled distribution (relative abundance by row) of each taxa across samples that were differentially associated with a group. Color intensity (warmth) represents relative abundances (dark blue = 0 to light yellow = 1). Only the highest taxonomic levels bearing any informative taxonomic labels and with an LDA eff size > 1 (log_10_ scale) are shown.

Figure 11 shows the distribution of the highest-level taxa that were differentially abundant in organs across the three regions and those differentials in regions across both organs. Taxa preferentially detected in intestine samples included genera *Vibrio*, *Propinigenium*, *Pseudoalteromonas*, and *Congregibacter*. The hepatopancreas-associated taxa included Proteobacteria, including genera *Methylobacterium*, *Acinetobacter*, *Pseudomonas*, and several families such as Bradyrhizobiaceae, Acetobacteraceae, Sphingomonadaceae, Enterobacteriaceae, and Xanthomonadaceae. Taxa from the Actinobacteria phylum that were differentially abundant and associated to this group included families Nocardioidaceae and Micrococcaceae, as well as genera *Corynebacterium* and *Propionibacterium acnes*. Genus *Bacillus* was also detected as differentially abundant and more prevalent in this group. A more significant number of differently abundant taxa were detected in organ groups than in region groups. Only order Cytophagales was found differentially more abundant in V3. In the case of V4, no associated taxa were found with LEfSe as statistically relevant. The V3V4 had differentially distributed taxa from various phyla, from families Rhodobacteraceae, Clostridiacea, Flavobacteriaceae, and Hyphomicrobiaceae.

We also analyzed differential taxa associated with the region-organ groups (Figure S7). These included family Sphingomonadaceae, associated with the V3-H, species Methylobacterium organophilum with the V4-H, as well as Prevotella copri, and genus Bacteroides. V4-I had a more significant prevalence of Cyanobacteria/Chloroplasts and Vibrio. Differentially abundant taxa associated with V3V4-H included the Caulobacteraceae and Burkholderiales families and with V3V4-I genus Propionigenium and Archea from the Halobacteriacea family.

Finally, independent organ and region comparisons were carried out to detect taxa that may show a differential distribution only seen when a group is isolated. As seen in Figure S8, there were fewer relevant features in these comparisons; thus, most were reported with uninformative labels. Of the hepatopancreas samples (Figure S8a), only Propionibacterium acnes had a stronger association with the V3 region. Also, and undetermined species of Methylobacteriaceae was also prevalent but not exclusive to V3, as it also was found in V4. It seems this is prevalent at a higher taxonomic resolution in V4 as Methylobacterium was reported to be overrepresented. As seen in the heatmap, most only Stramenopiles and possibly Burkholderiales were strongly associated with the V3V4 region, although the other four taxa were detected to be differentially abundant in that region. Intestine samples only suggested Vibrio as an interesting V4 associated-taxa, but this was also present in the V3V4 region (Figure S8b).

The family Vibrionaceae was found in V3, which suggests that this region has, in fact, a lower resolution for this particular genus (truncating it to family level). Comparisons of the organ-specific features in the different independent regions showed some similarities (Figure S8c–e), such as the prevalence of Stramenopiles in the intestine samples, in all three regions, and enriched Vibrionaceae in V3-I, comparable to enriched Vibrio in V4-I, possibly related to differences in resolution as well. A similar observation was made between the Methylobacteriaceae in V3-H, similar to the enrichment of Methylobacterium organophylum in V4. These differences in resolution may be mainly to the total sequence length of each region.

## 4. Discussion

The shrimp has become the most valuable traded marine product in the world, and its microbiota plays an essential role in its development and overall health. One of the key aims of this work was to investigate whether smaller and less costly to sequence regions of the 16S rRNA gene provided enough information for accurately describing shrimp microbiota. In this matter, we evaluated differences in the composition and abundance of the intestinal microbiota of *L. vannamei* using V3V4 amplicons, when compared against the shorter V3 and V4 individual regions.

### 4.1. Experimental and Analysis Considerations

According to the National Institute of Health (NIH) in the U.S., average sequencing costs have reduced exponentially following the adoption of high-throughput technologies, dropping from $898.90 to $0.015 USD per Mb over a decade (July 2005 to July 2015) [44]. However, sequencing costs are still a significant hurdle in most under developed countries, where the majority of shrimp farms are located, and much of the research is affected by international prices to import the sequencing consumables and equipment [1,45]. Further, most contemporary shrimp microbiota projects are accomplished with Illumina MiSeq platforms, which produces 20 Million reads spanning 500–600 bp inserts [20], characteristics that fit amplicons spanning two hypervariable regions for 16S profiling [7,12,13]. Alternative cost-effective sequencing platforms such as MiniSeq, producing up to 25 Million reads, may thus represent a step forward for shrimp research. However, these technologies tend to produce shorter reads (<300 nt), restricting their applications to the sequencing of a single 16S region for profiling such as V3 or V4.

Comparative analyses of the nine hypervariable regions in the 16S rRNA gene predate high-throughput sequencing and large-scale 16S profiling and have since shown critical differences in the maximum resolution achieved for monophyletic groups using different regions [46,47]. Although it is widely accepted that 97% identity clusters (OTUs) capture sequences of the same species, 94.5% is used for genus, 86.5% for family, 82.0% for order, 78.5% for class and 75.0% for phylum [48], the actual resolution depends on the phylogenetic congruence of the actual sequences in each cluster, ideally a monophyletic group [49]. Ultimately, there is no single set of primers that can completely identify the total microbial variation using isolated hypervariable regions of the 16S gene. In one of the largest *in silico* surveys to this date, Soergel and collaborators confirmed that microbial proportions varied widely between environments depending on the primer sets [50], and that the closest to “universal” coverage was achieved with sequences larger than 850 nt which are not currently viable in Illumina platforms [20]. Similarly, a study by Zhang and collaborators reported that amplicons spanning the V3 and V6 regions were the most variable and that the V3 and V4 regions capture the highest proportion of the total predicted variation in freshwater samples [51].

In the present study, the three regions studied (V3V4, V3, and V4) managed to capture most of the amplicon length variation that is expected for their corresponding sequenced inserts, as seen in the comparisons with the *in silico* sets (Figure 1). As expected, both MiniSeq derived sequencing sets (V3 and V4) presented a higher overall quality, but this is due to that error probabilities are more accumulated in the larger paired sequences produced by MiSeq (Figure S2). In this study, the length of the sequenced amplicon was the most critical limitation during sequence quality preprocessing and should be taken into account for further projects. When using 2 × 250 PE reads for analyzing V3V4 samples, the trimming of the 3′-end is critical for increasing the overlapping quality in the joining process. This overlap is ~30 nt long, and cannot withstand too stringent trimming parameters. The 2 × 150 PE fits right for the V3 region, resulting in a considerable overlap of ~>100 nt as the amplicon was shorter, and stringer trimming may not be too disruptive. In comparison, the success of using the 2 × 150 PE reads for the V4 region is highly dependent on the quality of the sequences. That is because the V4 amplicon size (~290) is very close to the size of the maximum insert size limit (<300), producing an overlap that ranges from 9 to 11 nt. As a result, any trimming step removing more than 5 nt irrevocably results in the loss of the overlapping region and, therefore, trimmed reads are not joinable.

Cluster grouping depends on sequence identity, and it is affected by the presence of the most divergent sequences. Clusters are thus formed differently whenever technical artifacts sequences are present (Figure S2). Although downstream frequency filters and rarefactions prevent the selection of most divergent clusters and possibly artifacts, these would not impact the taxonomic levels as much. Still, direct OTU comparisons are benefited by an initially randomized subsample to reduce this impact. In this regard, the randomized subsample of 16,793 also prevents that least abundant sequences influence the OTU formation before cross-region analyses and that each sample contributes with the same number of sequences to the clustering formation. We believe this is positive for the comparison as it standardizes the selection of the clusters. In this regard, previous studies also highlighted that the number of spurious OTUs increased with sequencing effort, suggesting that the comparison of communities should be made using an equal amount of sequences [52].

Of the single regions, V3 managed to capture a more varied spectrum than V4, possibly reflected by the shape of its length distribution showing two major peaks around 135 and 160 nt. This data is in accordance with the results of the large scale *in silico* survey by Soergel mentioned before, as simulated amplicons spanning the V3 region recovered a more significant proportion of the variability when compared to regions spanning the V4 region [50]. This ~25 nt variation between peaks has previously been attributed to changes in the RNA structure of the V3 region and has been reported to show associations to specific taxonomic classes [46]. For instance, most Cyanobacteria and Chloroflexi sequences have been associated with the small-size peak, whereas proteobacteria have been associated with the large-size peak [46]. This variation of the V3 region generates to the two populations in the longer V3V4 set (peaks at 405 and 427 nt), which is following the distributions and taxonomic observations in our BioSets (Figure 1).

Our results support that both the GreenGenes and silva databases are adequate for defining the shrimp microbiota. Furthermore, given that taxonomic labels vary among the different databases and phylogenetic topologies, making them not directly comparable between one another [53]. The number of assigned taxonomies in both databases confirmed to be mostly equivalent up to the family level (Figure 3). However, the silva database produced a more significant proportion of non-informative high-level taxa (i.e., uncultured and clade group tags), which led us to use GreenGenes for our analyses. Also, the GreenGenes database is still extensively employed, and it is the primary source for taxonomy-related methods in the QIIME2 analysis suite as of the date of this study [34,42].

Contrastingly, the *in silico* sets containing all the available amplicons for each reference database showed that the primers used in our study could virtually recover 99% of the OTUs with any of the three regions. Notably, when *in silico* amplicons were clustered, the V3 region generates less total OTUs due to its shorter length as compared to V4, while the V3V4 region, captures a more significant collection of OTUs. Interestingly, the shrimp microbiota was better captured by V3V4, followed by V3 and lastly by V4. Also worth noting, with our present data, four samples were enough to detect that the V3 region had more unique items than the V4 region (Figure 4b). As confirmed by the OTU recruitment analysis, due to the more considerable variability of longer sequences, using the V3V4 region requires a higher sequencing depth to release its full diversity potential. In contrast, most V3 and V4 samples had recruited the majority of their variation with less than 10,000 reads (Figure 4).

### 4.2. Microbiota Structure and Composition

The region followed by the organ drove the most pronounced variations in the beta-diversity. However, when the samples were independently analyzed for each region, the organ drove the OTUs variation. This is in accordance with the previous data observed that the hypervariable region showed a significant impact on the microbiota structure [14], but here, using the same experimental and bioinformatics process on the same set of samples.

The three analyzed regions assessed a higher OTU richness for the intestine samples when compared to the hepatopancreas (Figure 4 and Figure 9), which was following previous studies [7]. Additionally, the hepatopancreas shows a higher diversity (Shannon index), also independently of the region sequenced. These data suggest that there is a higher selective pressure on microbiota richness and diversity in hepatopancreas than in the intestine. In this regard, this behavior could be explained by the selective pressure of the organ, which produces immune molecules such as lectins, hemocyanin, ferritin, antibacterial proteins, proteolytic enzymes, and nitric oxide and may therefore prevent the establishment of some species [54].

The difference between expected (Chao1) and observed OTUs showed that the V3V4 region captured more of its expected variation (~85%) when compared to the single regions (see alpha rarefaction results). Intestine samples had more significant number of predicted OTUs (Figure 8a) regardless of the region, but these were more unevenly distributed (lower Shannon’s entropy; Figure 8b) than in hepatopancreas. Regarding taxonomic composition (Figure 5), the V3V4 region reaches an optimal genus-level resolution, whereas single regions perform better at the family level. This limitation is probably due to the shorter length of the sequences as it has been reported that 96 nt are sufficient to provide 80% genus classifications [50].

At the phylum level and similar to previous studies, Proteobacteria was the most abundant taxa in most samples, followed by a substantial prevalence of Cyanobacteria in the intestine samples [7,10,14,55]. Both phyla have been reported in the natural bacterioplankton of the intestine of shrimp captured from lakes and rivers [17]. It is also noticeable that even at this level, the V3V4 sets were far more diverse, recovering additional taxa from the flagellated aquatic phyla Planctomycetes. Phyla Actinobacteria and Firmicutes were more abundant in the hepatopancreas as compared to the intestine, especially in the V3 region samples.

The Stramenopiles were highly abundant in the shrimp intestines, ~19% (Figure 7 and Figure 11). At the family level, homologs of Cyanobacteria were classified as Stramenopiles chloroplasts, which has also been reported in other studies [7,17,56]. This is a common observation in the microbiota of aquatic niches, as chloroplasts from eukaryotic algae are considered to have originated from free-living photosynthetic Cyanobacteria [56]. It has been documented that their shared evolutionary history makes it challenging to have confidence in separating these two 16S and 18S sources [57]. Bioinformatic protocols commonly include steps to remove sequences marked as chloroplast [9]. Still, due to their prokaryotic origins, this may also filter actual cyanobacteria, some of which are dominant oxygenic phototrophs in the tropical and subtropical regions [58]. Also, since cyanobacteria occur naturally in aquaculture of shrimps, it is probably best not to remove them unless they can be safely discarded as chloroplasts. Thus, we decided to maintain the Stramenopiles_cloroplast taxa in our set. Interestingly, both types of eukaryotic Stramenopiles or Stramenopiles_cloroplast classified as Cyanobacteria have been found using 18S and 16S approaches in shrimp intestine microbiota in higher abundances, suggesting that this family could be part of the typical shrimp diet [59,60,61]. Although it is out of the scope of this work, a way to tackle the problem might be to use a dedicated cyanobacteria database 16S database such as the CyanoDB, to discard those that are 97% identical to known chloroplast sequences in the regions of interest [62].

Several of the reported Proteobacteria were identified as homologs to the ubiquitous, soil, and water-dwelling family Methylobacteraceae [63], a methylotrophic group that was prominently found in hepatopancreas samples of V3 and V4. Also importantly, the V3V4 region was far more diverse, and the top 50 represented ~90% of the total families. Other families with intra-group variability include Fusobacteriaceae, more abundant in the intestine, which is dedicated to extract energy through the fermentation of a variety of carbohydrates, amino acids and peptides [64], and the chemo-organotrophic Caulobacteraceae family, more prominent in hepatopancreas with a characteristic alkaline phosphatase activity [65], consistent with the organ function.

After a LEfSe analysis (Figure 11), several species were detected as differentially abundant among groups. Notably, all three regions detected Cyanobacteria and Vibrio, one of the most commonly studied [7] and *Propionigenium*, a strictly anaerobic genus dependent on decarboxylation of succinate to propionate [66] as differentially abundant taxa in the intestine. In the hepatopancreas, *Methylobacterium*, and *Propionigenium* were more commonly found in V4 and V3 samples, respectively, although due to their ubiquitous nature, they have been commonly regarded as potential contaminants [67]. However, the fact that only these two bacteria were abundant in hepatopancreas discards the potential contamination by reagents kits to extract DNA because the DNA of all organ samples was isolated using the same kit. The effect size of other taxa was far less pronounced (LDA < 2), and no differentially abundant taxa were associated with or organ division, although several items were independently associated with sequenced regions, as seen in Figure S6. Interestingly, in a recent meta-analysis that include data from multiple 16S rRNA regions [14], our group also detected Vibrio as differentially abundant taxa in the intestine, as well as Acinetobacter and Pseudomonas in the hepatopancreas (Figure 11), suggesting that these taxa could be considered as markers for their respective organs independently of the analyzed region.

As previously observed in other studies, the Adonis test shows that technical factors such as the hypervariable region have the most significant impact in the β-diversity analysis [14], followed by biological aspects such as the organ. From this comparison, the V3V4-I and V3V4-H sets were shown to be more homogeneous (Figure 9). This data was similar to that observed with region grouping, in which the V3 and V4 were more similar by organ than by region. This means that although all three regions can be used to capture the global OTU differences between organ groups effectively, single regions (V3 and V4) produce more differentiated organ clusters probably due to the shorter length of individual regions limits the resolution ignoring part of the bacterial spectrum, while the more extended V3V4 region reveals more homogeneity between organs. To test this, we added phylogenetic information (UniFrac), which allows all three regions to show organ differences more clearly. This, however, can only be carried out one region at a time as sequences are different. The observations from the ordination methods were confirmed by the sample correlation analyses, which also supported the organ similarity of the V3 and V4 at the level of the genus (Figure 10). In terms of distribution, the V3V4 was more similar between the two organs. Contrastingly, all V3 and V4 samples showed higher correlations with samples from the same organ than from the same region.

Few taxa were reported to be associated with specific region groups by the LEfSe analyzes (considering both organ groups), most of them were detected exclusively in the more diverse V3V4 region. However, different taxa were associated with each organ-group cluster: Vibrio and Stramenopiles, mentioned above, were more abundant in intestine samples of the V4 region but not in the hepatopancreas samples. The V3-H allowed for the preferential detection of Geobacillus, Sphingomonadaceae, and Rhodospirillales, the V3-I group of Pseudoalteromonadaceae, the V-H group of Methylobacterium organophilum, Prevotella copri, Bacteroides and Verrucomicrobiaceae. V3V4 preferentially detected Mollicutes, Caulobacteraceae and Burkholderiales, and V3V4-I Propionigenium, Synechococcus, and Halobacteriaceae. Analyzing isolated organs and regions (without considering other groups) revealed that taxa differences that drives group separation were greatly influenced by uneven sample resolution, especially in the V3 region, which forms larger clusters (Figure S8).

Regarding our study’s limitations, in an ideal setup, all three sets would be sequenced, not only in the same platform and sequencing service but also in the same equipment to avoid part of the technical biases. Yet, the PCR-related differences such as primer degeneracy, melting temperatures, and thermocycler protocols would still impact actual comparisons. Despite this, library preparation was standardized for all three sets using the same Illumina adapters. In our data, we found the sequencing platform only contributes to the composition with R^2^ = 0.134 and abundance variation with R^2^ = 0.172, which were lower than observed for organs and regions. However, this lower effect also can be due to the contribution of organs and regions, which cannot be separated from the sequencing platform in our sequenced dataset. On the other hand, it has been reported that different sequencing platforms impact the microbiota analysis [14,68]. However, the comparisons were between totally different technological, experimental, and sequencing platforms such as 454 (Roche), Ion torrent (Invitrogen), MiSeq (Illumina), and HiSeq (Illumina). Additionally, a meta-analysis of shrimp microbiota discovered that despite the effects of the technical factors, the impact of biological factors could also be observed even using very different sequencing platforms [14]. Recently, a study compared MiniSeq vs. MiSeq by sequencing the same hypervariable region, and they found no significant effect between these two very similar sequencing platforms [69]. All the data mentioned above, suggests that although our experimental design does not allow us to what is the impact of the sequencing platforms in our microbiota analysis, if there were, it would be marginal. Although this unevenly affects V3V4 set in terms of quality due to its longer insert size, we have applied a stringent quality controls and filtering steps downstream to enable a balanced comparison between sequencing platforms and amplicon sets.

## 5. Conclusions

Considering single hypervariable regions, V3 is more variable in sequence lengths compared to the V4 region, which reflects on a wider variety of unique OTUs, regardless of the reference database that was used for clustering. Both regions have an optimal resolution at the family level, and most families also have informative genera. On the other hand, the V3V4 region produces more significant sequence variability than single regions, doubling the taxa recovered in most taxonomic levels and reaching an optimal genus-level resolution. Both the GreenGenes and silva databases are suited for the analysis of shrimp microbiota, producing a similar number of taxa that is mostly the same up until the family level. Still, each has some limitations, in silva, the genus and species levels bear a large proportion of non-informative tags, whereas GreenGenes have some outdated entries.

Regarding microbial diversity, the V3V4 region had a higher number of unique OTUs, and they were more evenly distributed (richness and entropy). Samples in V3 and V4 regions had fewer taxa and were more unevenly distributed, but were more congruent with one another by organ. In this regard, the sample correlation confirmed that the V3 and V4 samples were more similar depending on the organ than on the region, whereas V3V4 samples formed a more consistent group that mainly depends on the region. In terms of taxonomy, V3V4 samples recovered a much more varied collection when compared to V3 and V4. Proteobacteria was the most abundant phyla overall. Detection of Cyanobacteria was more common in the intestine samples, whereas Firmicutes and Actinobacteria were more prevalent in hepatopancreas samples. Larger effect sizes (LDA > 2) were reported in organ-associated taxa but not with regions. Finally, the β-diversity analysis showed that the hypervariable region explained more of the variation than the organ. However, at the OTU level, there are distinct clusters that differentiate the intestine from the hepatopancreas independently of the analyzed region.

To summarize, all three regions can be successfully used to study *L. vannamei’s* microbiota, depending on the objective of the study. V3 captures a broader spectrum of diversity than V4. Thus, for an exploratory study, analyzing a single region such as V3 can provide enough information at a lower cost and faster sequencing times to describe the composition and structure of the shrimp microbiota. Farther, spanning the V3V4 region, provides a more in-depth analysis in terms of OTU clustering and taxonomic resolution, due to its longer amplicon size, which can be necessary to obtain more detailed information about the microbial communities present; however, this it is more expensive in sequencing cost. To further improve this type of analysis and fully define the total shrimp microbiota, a whole-genome analysis approach (shotgun sequencing) could help to explore the functional differences provided by different regions, a valuable future insight for the study of shrimp microbiota.

## Supplementary Materials

The following are available online at https://www.mdpi.com/2076-2607/8/1/134/s1, Table S1 Sequence preprocessing totals, Figure S1: Workflow summary, Figure S2: Sequence quality of each amplicon set, Figure S3: Percentage of items grouped into OTUs in the gg97 and silva97 BioSets, Figure S4: Correlation of total unique OTUs between gg97 and silva97 BioSets, Figure S5: Family relative abundance per sample, Figure S6: PCoA of phylogentetically-defined differences in OTU composition and abundance by region, Figure S7: Distribution of differentially abundant multi-level taxa selected by the LDA Effect Size (LEfSe) associated to region-organ sample groups, Figure S8: Distribution of differentially abundant multi-level taxa selected by the LDA Effect Size (LEfSe) associated to split region and organ groups. Supplementary material with information on data processing and analysis.
