# Supplementary material for: Doing More with Less: A Comparison of 16S Hypervariable Regions in Search of Defining the Shrimp Microbiota
Source: Microorganisms. 2020 Jan 17;8(1):134. doi: 10.3390/microorganisms8010134 (PMC7022540; doi:10.3390/microorganisms8010134)
Supplement: Supplementary file 1 [file microorganisms-08-00134-s001.zip › microorganisms-670933 suppl for final 1.17/microorganisms-670933 suppl for proof copy.pdf]

Supplementary Material

Detailed Sequencing Set Preprocessing

The following section describes the preprocessing of the sequencing data in detail, including non-default parameters used for enabling a balanced comparison between the different platforms and amplicon sets. Short-reads were eliminated before any processing steps for each set, considering the average size of each hypervariable region and the length of the overlap between the paired-ends as follows: V3 < 120 bp, V4 < 150 bp, V3V4 < 170 bp to recover joinable insert sequences. Short reads in all three sets were removed considering the theoretical length of the amplicons per region. Reads in the V3 set that were shorter than 102 were removed, keeping those with a theoretical overlap >~10 nt of the shortest references for this region in a 2 × 150 run. Reads in the V4 set that were shorter than 150 nt (full-length sequences) were removed as the theoretical overlap with 2 × 150 PE reads is expected to be ~9 nt. This high-stringency filter was selected because sequences shorter than this cutoff would result in no overlap in V4 PE reads, rendering them not joinable (the V4 region insert is ~290 bp long). Furthermore, MiniSeq platforms produce >80% bases higher than Q30 at 2 × 150 bp; hence they can handle a more stringent filter. Reads in the V3V4 set that were shorter than 170 were removed as a conservative approach as MiSeq is expected to produce >75% bases higher than Q30 at 2 × 250 bp. The minimum length for the V3V4 region in 2 × 250 PE sets to include an overlap would, in fact, allow up to a ~210 nt filter. Still, it would negatively impact downstream cleansing steps due to the expected quality drops in the 3′ regions that are intrinsic of Illumina's sequence-by-synthesis approach. The corresponding sequence of 16S amplification primers were removed from the 5′-end of sequences using Cutadapt v2.0, considering primer sequence ambiguity and enabling partial matches, ensuring a sequence probability by chance < 9.54×10^{-7}, which was adjusted to each of the sequencing sets.

For the V3 set, primer 338F sequence (5′-ACTCCTACGGGAGGCAGCAG-3′) was trimmed (e = 0.2; o = 14) from R1 (forward sequence of each pair), and primer 533R sequence (5′-TTACCGCGGCTGCTGGCAC-3′) was trimmed (e = 0.25; o = 14) from R2 (reverse sequence of each pair). For V4, primer 515F sequence (5′-GTGCCAGCMGCCGCGGTAA-3′) was trimmed (e = 0.2; o = 14) from R1, and primer 806R sequence (5′-GGACTACHVGGGTWTCTAAT-3′) was trimmed (e = 0.25; o=14) from R2. For the V3V4 set, the primer 341F sequence. (5′-CCTACGGGNGGCWGCAG-3′) was trimmed (e = 0.4; o = 14) from R1, For the V3V4 set, and the primer 805R sequence (5′-GACTACHVGGGTATCTAATCC-3′) was trimmed (e = 0.4; o = 17) from R2. Existing partial sequences of Illumina Nextera adapters were removed from the 3′-end of all sets using the sequences of non-biological constructs with the structure: 16S primernextera_adapter-index-sequencing_primer, allowing for a variable length. In the V3 set, construct 5′-CAGCAGCCGCGGTAACTGTCTCTTATATACATCTCCGAGCCCACGAGACN{8}ATCTCGTATG CCGTCTTCTGCTTG-3′ was trimmed (e = 25; o = 21) from R1, and construct 5′-CCTCCCGTAGGAGTCTGTCTCTTATACACATCTGACGCTGCCGACGAN{8}GTGTAGATCTCG GTGGTCGCCGAATCATT-3′ was trimmed (e = 25; o = 21) from R2. In V4, construct 5′-ATTAGAWACCCBDGTAGTCCCTGTCTCTTATACACATCTCCGAGCCCACGAGACN{8}ATCTC GTATGCCGTCTTCTGCTTG-3′ was trimmed (e = 25; o = 21) from R1, and construct 5′-TTACCGCGGCKGCTGGCACCTGTCTCTTATACACATCTGACGCTGCCGACGAN{8}GTGTAGA TCTCGGTGGTCGCCGAATCATT-3′ was trimmed (e = 25; o = 21) from R2. In V3V4, construct 5′-GGATTAGATACCCBDGTAGTCCTGTCTCTTATACACATCTCCGAGCCCACGAGACN{8}ATCT CGTATGCCGTCTTCTGCTTG-3′ was trimmed (e = 0.15; o = 17) from R1, and 5′-CTGCSGCCNCCCGTAGGCTGTCTCTTATACACATCTGACGCTGCCGACGAN{8}GTGTAGATC TCGGTGGTCGCCGTATCATT-3′ was trimmed (e = 15; o = 17) from R2. The correct trimming of the

16S primers was confirmed by flanking sequence analysis with prinseq. All PE reads were subjected to the following streamlined cleansing protocol using Prinseq: 1). Filter reads with low entropy, including homopolymers and spurious repeats (entropy < 68%). 2) Trim low quality 3′-ends of all sequences based on Phred quality scores (5 nt window, step = 3, type = mean, q = 17). 3) Trim low quality 5′-end of reads (5nt window, step = 3, type = min, q = 20). 4) Filter sequences of low average quality (q = 22). 5) Further trim the 3′-end with a more stringent window (3 nt window, step = 3, type = mean, q = 20). Only sequences in which both pairs were conserved after cleaning were considered for downstream steps.

All pairs of clean PE sequences were subsequently joined with COPE v1.2.5. Parameters were adjusted to each region, depending on the expected size of the insert. For the V3, an overlap of 20-135 nt was allowed with an insert of an average 145 and match ratio cutoff (-c) of 0.25. For V4, overlap 9–11 nt, insert = 250, c = 0.25. For V3V4, overlap = 30–250, insert = 420, c = 0.25. To prevent spurious sequences from biasing OUT formation (by deviating the correct selection of the cluster centroids), all samples were resampled to 16,793 reads with seqtk, reflecting the total abundance of the smallest sample in the whole set, given by a V3V4 sample from the hepatopancreas group). This was carried out with a different seed to ensure randomness, which was set to their total number of reads per sample. Resampling was done randomly and without replacement using the joined reads. Lengths of all sequences were evaluated to assess the successful recovery of each region. The in house developed scripts are available at https://github.com/8aLab/2020-microorganisms-hypervariable_regions.
