# Supplementary material for: Doing More with Less: A Comparison of 16S Hypervariable Regions in Search of Defining the Shrimp Microbiota
Source: Microorganisms. 2020 Jan 17;8(1):134. doi: 10.3390/microorganisms8010134 (PMC7022540; doi:10.3390/microorganisms8010134)
Supplement: Supplementary file 1 [file microorganisms-08-00134-s001.zip › microorganisms-670933 suppl for final 1.17/Supplementary_figures for proof.pdf]

## Supplementary Figures

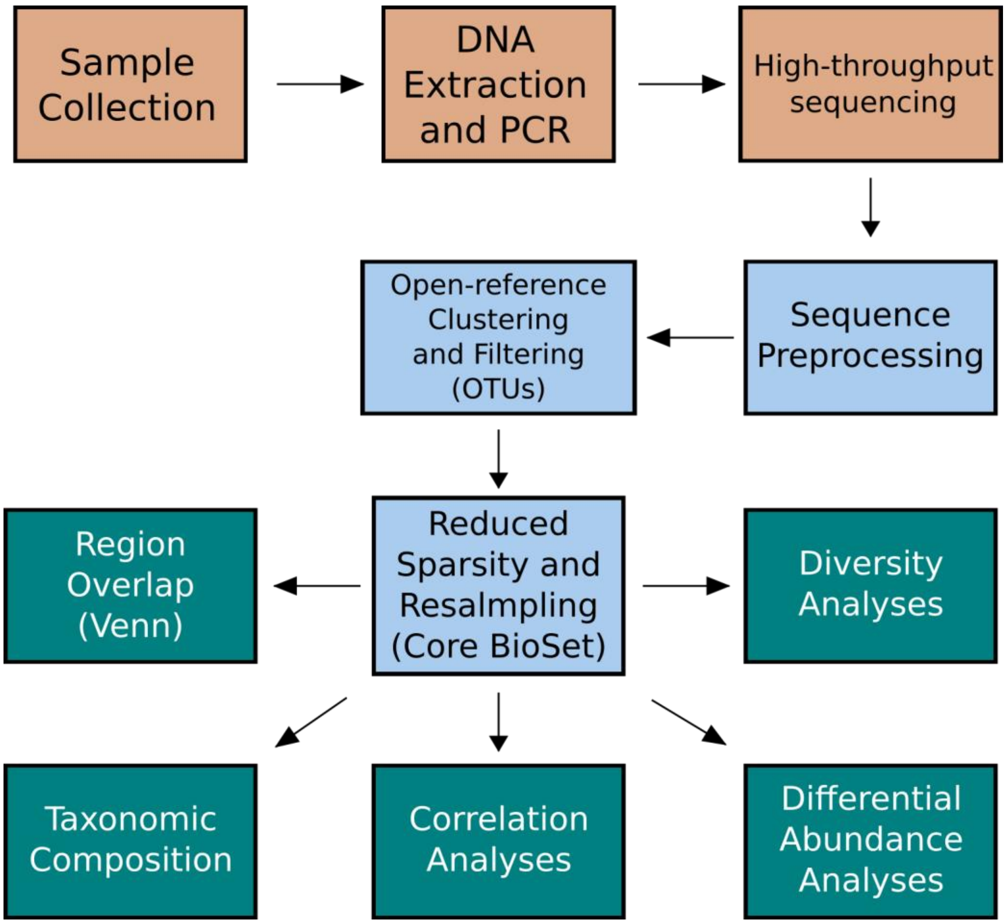


**Figure S1.** Workflow summary. Wet lab (experimental) procedures are shown in the brown panels. Blue panels show bioinformatic methods and green ones show the different set of analyses carried out for them.

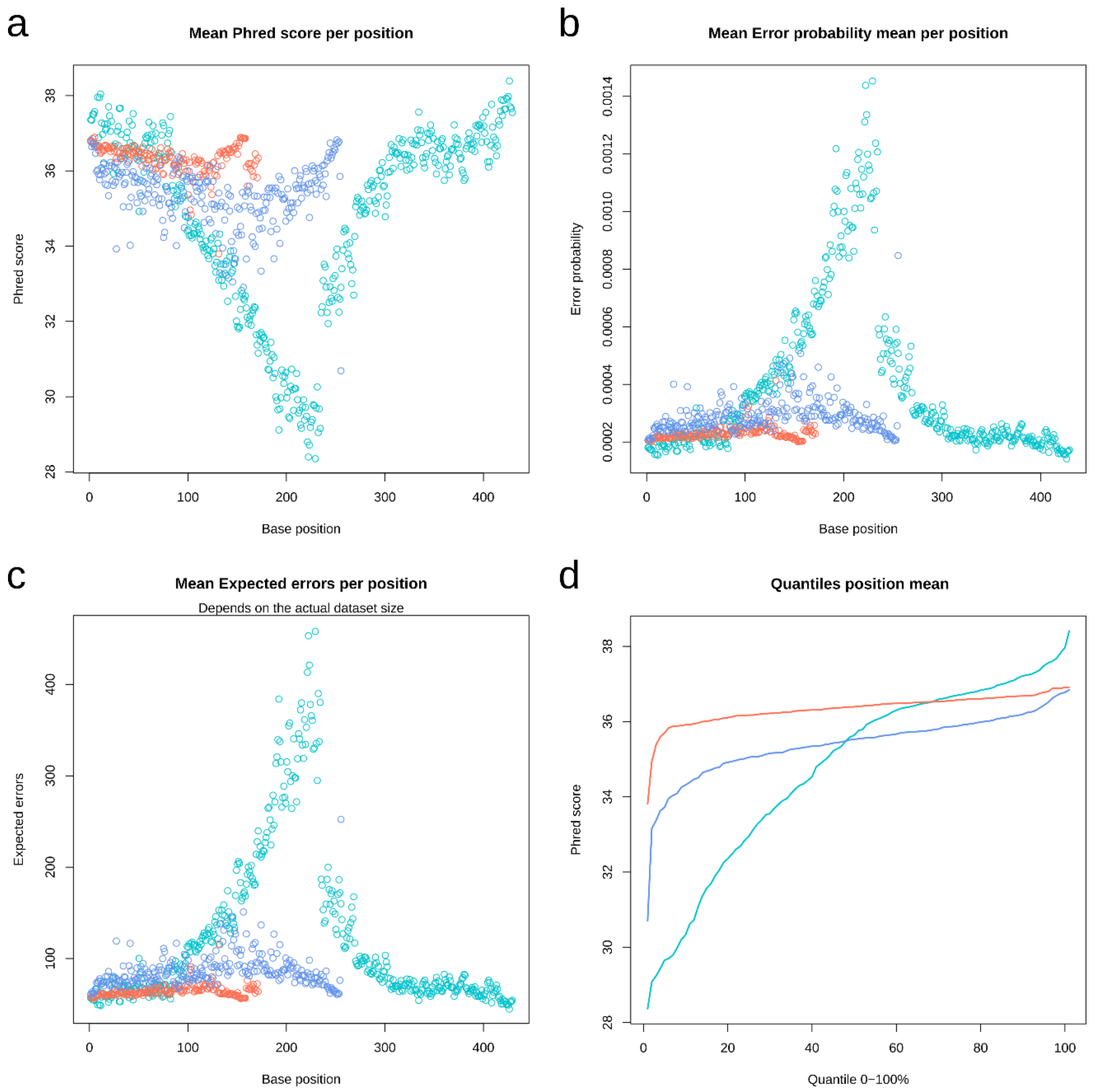


**Figure S2.** Sequence quality of each amplicon set. Reads are presented after sample preprocessing and paired-end joining. V3 is presented in coral, V4 in blue and V3V4 in aquamarine. (**a**) Average Phred quality scores at each position. (**b**) Error probability associated to the mean score in panel a. (**c**) Expected errors calculated based on the error probabilities and the total reads in each region set. (**d**) Position quality mean quantiles 1–100%.

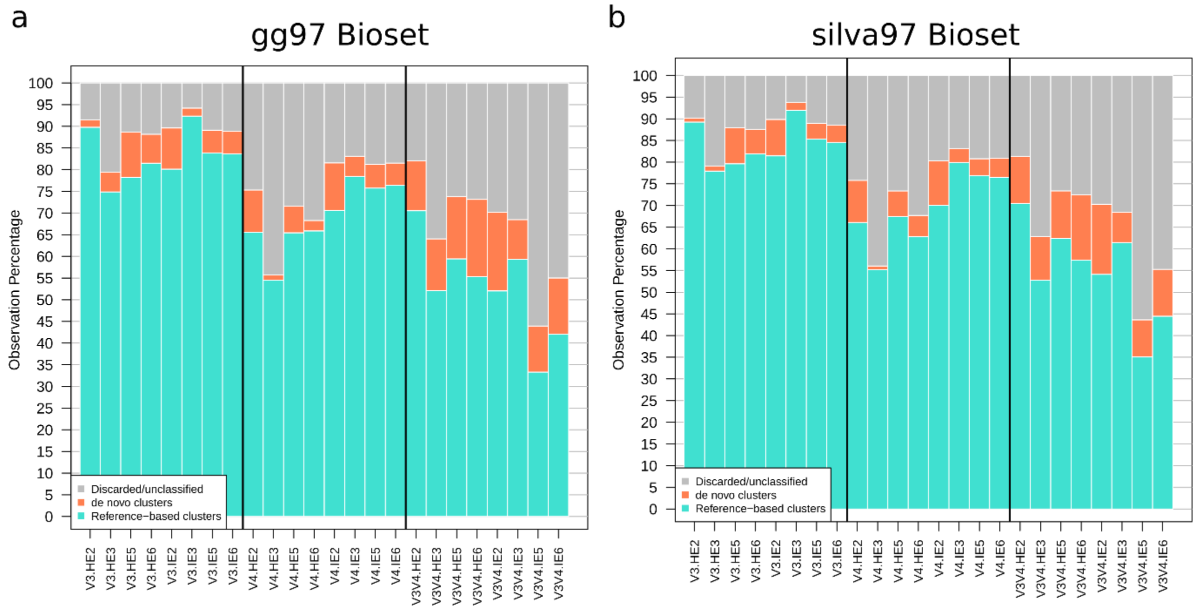


**Figure S3.** Percentage of items grouped into OTUs in the gg97 (**a**) and silva97 (**b**) BioSets. Barplots are shown for each sample reflecting the proportion of input reads that were assigned to valid clusters. Samples are grouped by region (left: V3; center: V4; right: V3V4). Type of OTUs are colored by reference-based clusters (turquoise) and de novo clusters (coral). Gray bars represent features that were discarded or non assigned to any clusters.

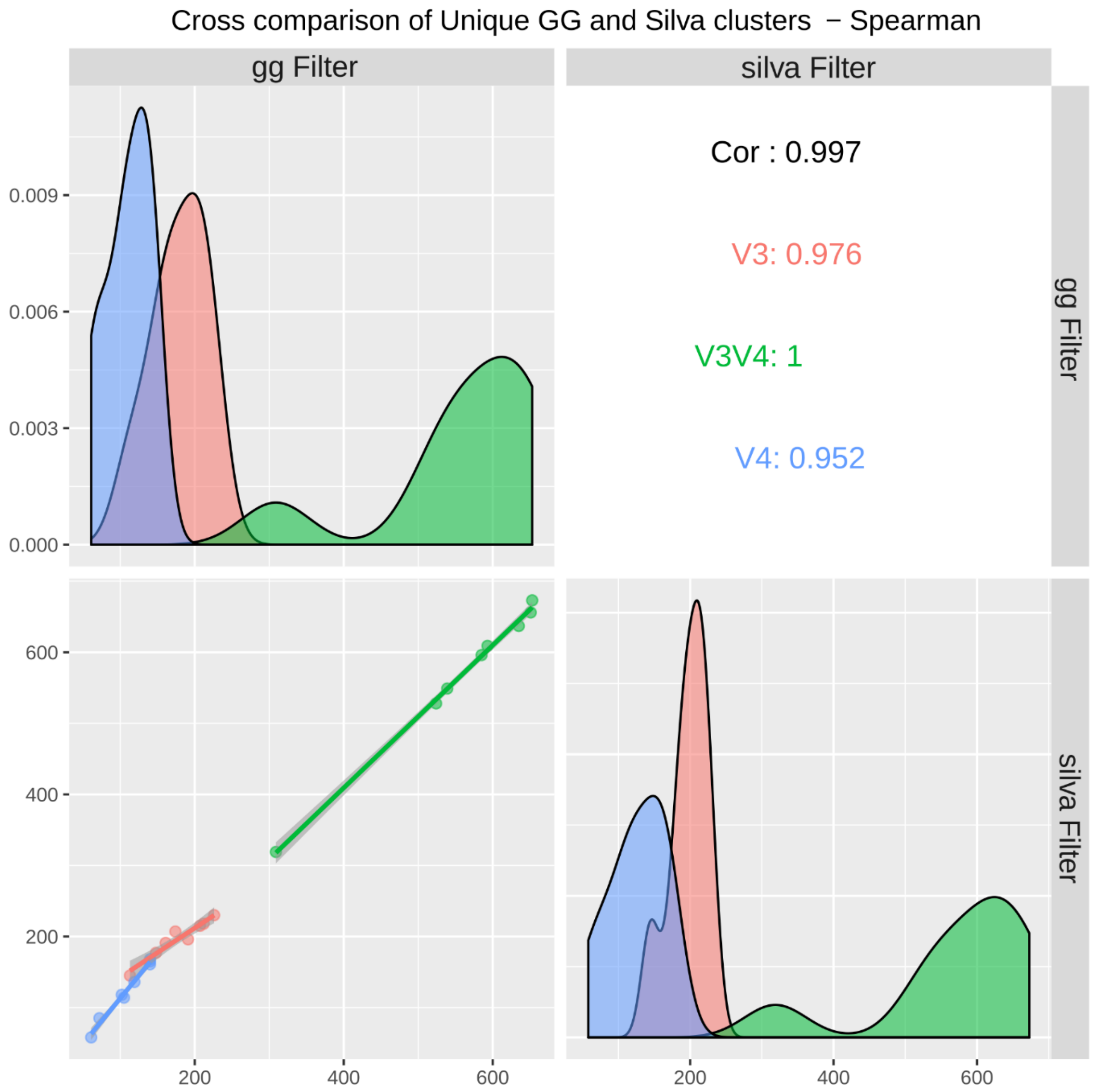


**Figure S4.** Correlation of total unique OTUs between gg97 and silva97 BioSets. All comparisons are broken down by region (pink: V3, blue: V4, green: V3V4). Density plots depict the distribution of total items per sample in the three regions. The linear regression functions depict the relation of both sets by comparing each region subset. Correlations for the whole table and the three regions are included.

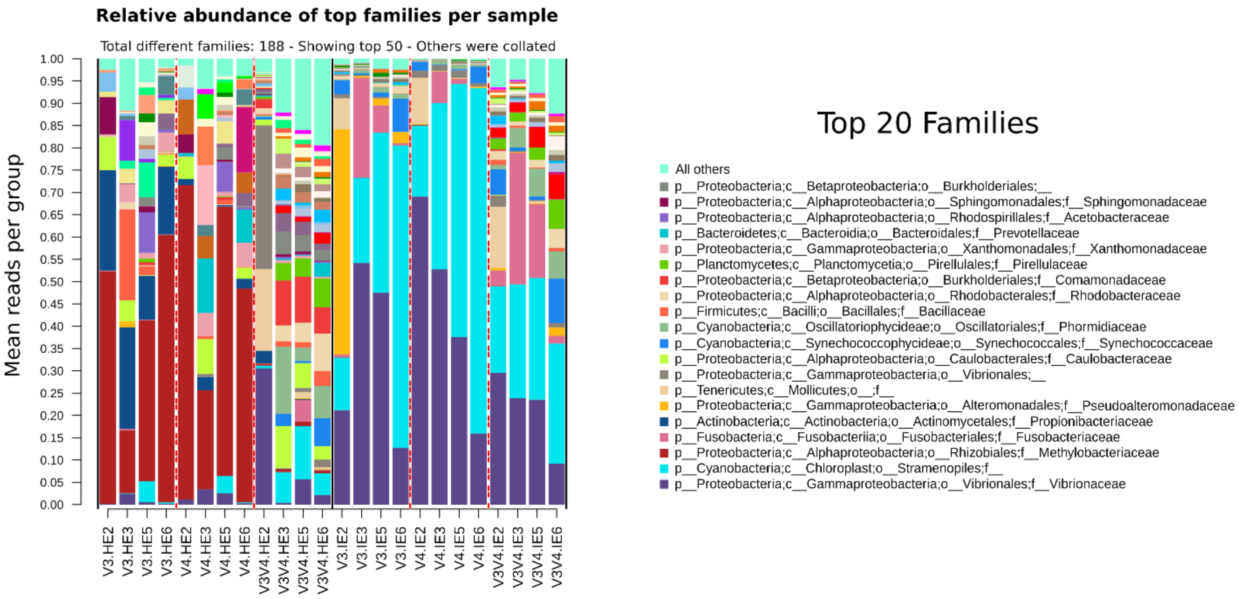


**Figure S5.** Family relative abundance per sample. Families are presented in order of their total abundance in the table. Only top 50 are shown and labels for the top 20 are presented; the sum or the rest are shown as All others. Samples are presented by organ-region groups.

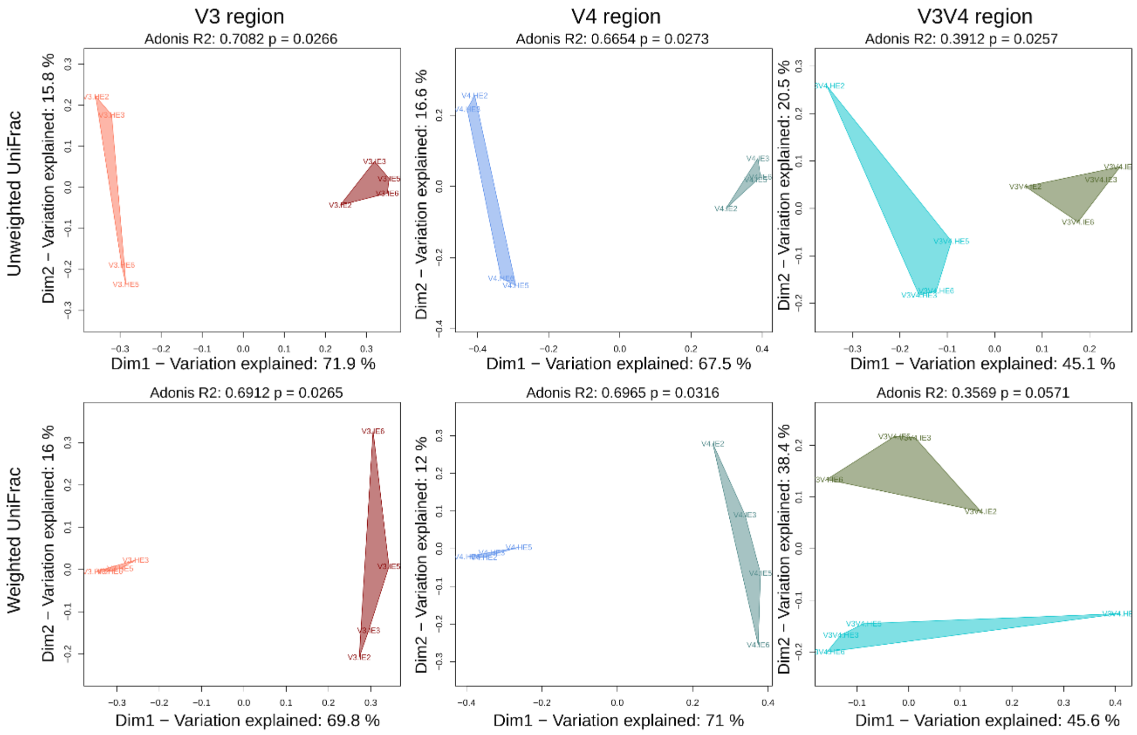


**Figure S6.** PCoA of phylogentetically-defined differences in OTU composition and abundance by region. PCoA from weighted and unweighted UniFrac of the three region sample subsets. Phylogenetic information was only relevant when analyzed per region since representative sequences were primer-specific. (Legend): V3-H: hepatopancreas samples in the V3 region subset, V4-H: hepatopancreas samples in the V4 region subset, V3V4-H: hepatopancreas samples in the V3V4 region subset, V3-I: intestine samples in the V3 region subset, V4-I: intestine samples in the V4 region subset, V3V4-I: intestine samples in the V3V4 region subset.

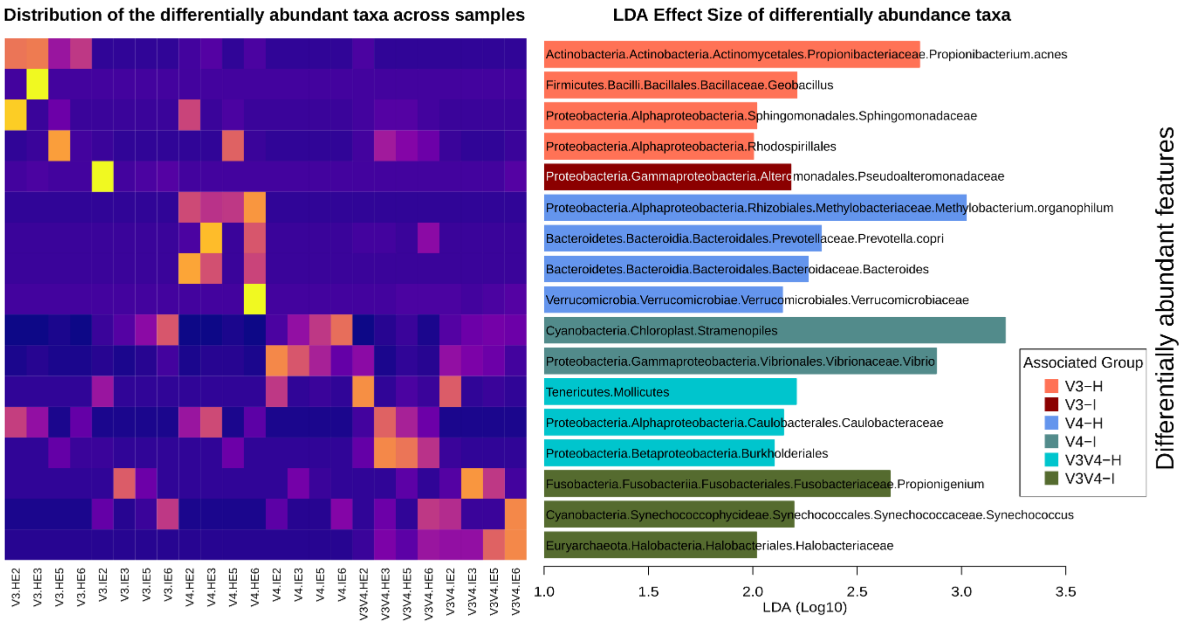


**Figure S7.** Distribution of differentially abundant multi-level taxa selected by the LDA Effect Size (LEfSe) associated to region-organ sample groups. The heatmap represents the scaled distribution of each taxa across the samples, that is differentially associated to a group by their LEfSe. Shown taxa are preferentially detected in the indicated region-organ group. Color intensity (warmth) represents relative abundances (dark blue = 0 to light yellow = 1). LDA is on a log10 scale and features with an effect size >2 are shown. Only the highest taxonomic levels bearing any informative taxonomic labels are shown (from phylum to the highest level available for each taxon using the greengenes' nomenclature).

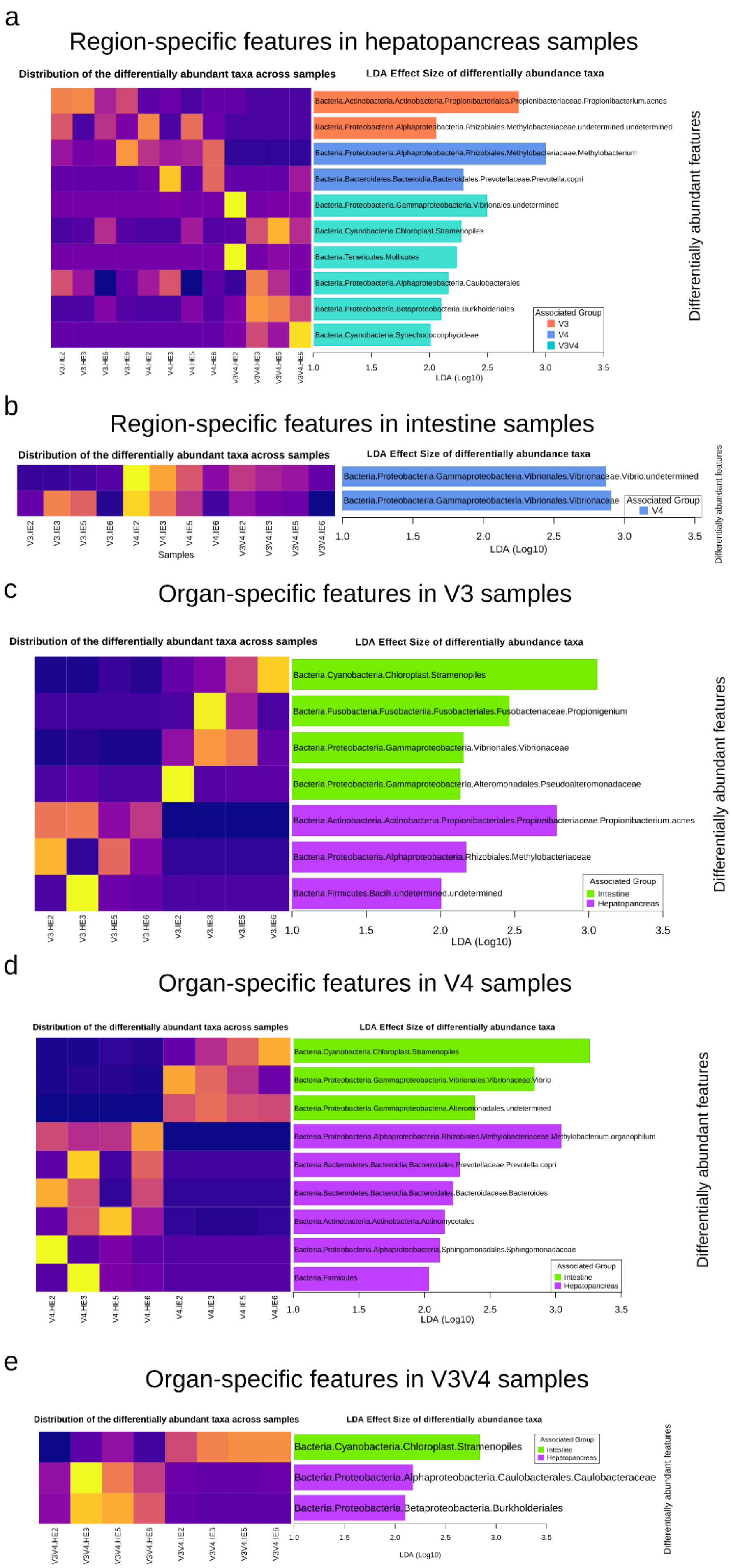
a
Region-specific features in hepatopancreas samples
Distribution of the differentially abundant taxa across samples
LDA Effect Size of differentially abundance taxa
Bacteria.Actinobacteria.Actinobacteria.Propionibacteriales.Propionibacteriaceae.Propionibacterium.acnes
Bacteria.Proteobacteria.Alphaproteobacteria.Rhizobiales.Methylobacteriaceae.undetermined.undetermined
Bacteria.Proteobacteria.Alphaproteobacteria.Rhizobiales.Methylobacteriaceae.Methylobacterium
Bacteria.Bacteroidetes.Bacteroidia.Bacteroidales.Prevotellaceae.Prevotella.copri
Bacteria.Proteobacteria.Gammaproteobacteria.Vibrionales.undetermined
Bacteria.Cyanobacteria.Chloroplast.Stramenopiles
Bacteria.Tenericutes.Mollicutes
Bacteria.Proteobacteria.Alphaproteobacteria.Caulobacterales
Bacteria.Proteobacteria.Betaproteobacteria.Burkholderiales
Bacteria.Cyanobacteria.Synechococcophycideae
Associated Group
V3
V4
V3V4
V3.HE2 V3.HE3 V3.HE5 V3.HE6 V4.HE2 V4.HE3 V4.HE5 V4.HE6 V3V4.HE2 V3V4.HE3 V3V4.HE5 V3V4.HE6
1.0 1.5 2.0 2.5 3.0 3.5
LDA (Log10)
Differentially abundant features
b
Region-specific features in intestine samples
Distribution of the differentially abundant taxa across samples
LDA Effect Size of differentially abundance taxa
Bacteria.Proteobacteria.Gammaproteobacteria.Vibrionales.Vibrionaceae.Vibrio.undetermined
Bacteria.Proteobacteria.Gammaproteobacteria.Vibrionales.Vibrionaceae
Associated Group
V4
V3.IE2 V3.IE3 V3.IE5 V3.IE6 V4.IE2 V4.IE3 V4.IE5 V4.IE6 V3V4.IE2 V3V4.IE3 V3V4.IE5 V3V4.IE6
Samples
1.0 1.5 2.0 2.5 3.0 3.5
LDA (Log10)
Differentially abundant features
c
Organ-specific features in V3 samples
Distribution of the differentially abundant taxa across samples
LDA Effect Size of differentially abundance taxa
Bacteria.Cyanobacteria.Chloroplast.Stramenopiles
Bacteria.Fusobacteria.Fusobacteriia.Fusobacteriales.Fusobacteriaceae.Propionigenium
Bacteria.Proteobacteria.Gammaproteobacteria.Vibrionales.Vibrionaceae
Bacteria.Proteobacteria.Gammaproteobacteria.Alteromonadales.Pseudoalteromonadaceae
Bacteria.Actinobacteria.Actinobacteria.Propionibacteriales.Propionibacteriaceae.Propionibacterium.acnes
Bacteria.Proteobacteria.Alphaproteobacteria.Rhizobiales.Methylobacteriaceae
Bacteria.Firmicutes.Bacilli.undetermined.undetermined
Associated Group
Intestine
Hepatopancreas
V3.HE2 V3.HE3 V3.HE5 V3.HE6 V3.IE2 V3.IE3 V3.IE5 V3.IE6
1.0 1.5 2.0 2.5 3.0 3.5
LDA (Log10)
Differentially abundant features
d
Organ-specific features in V4 samples
Distribution of the differentially abundant taxa across samples
LDA Effect Size of differentially abundance taxa
Bacteria.Cyanobacteria.Chloroplast.Stramenopiles
Bacteria.Proteobacteria.Gammaproteobacteria.Vibrionales.Vibrionaceae.Vibrio
Bacteria.Proteobacteria.Gammaproteobacteria.Alteromonadales.undetermined
Bacteria.Proteobacteria.Alphaproteobacteria.Rhizobiales.Methylobacteriaceae.Methylobacterium.organophilum
Bacteria.Bacteroidetes.Bacteroidia.Bacteroidales.Prevotellaceae.Prevotella.copri
Bacteria.Bacteroidetes.Bacteroidia.Bacteroidales.Bacteroidaceae.Bacteroides
Bacteria.Actinobacteria.Actinobacteria.Actinomycetales
Bacteria.Proteobacteria.Alphaproteobacteria.Sphingomonadales.Sphingomonadaceae
Bacteria.Firmicutes
Associated Group
Intestine
Hepatopancreas
V4.HE2 V4.HE3 V4.HE5 V4.HE6 V4.IE2 V4.IE3 V4.IE5 V4.IE6
1.0 1.5 2.0 2.5 3.0 3.5
LDA (Log10)
Differentially abundant features
e
Organ-specific features in V3V4 samples
Distribution of the differentially abundant taxa across samples
LDA Effect Size of differentially abundance taxa
Bacteria.Cyanobacteria.Chloroplast.Stramenopiles
Bacteria.Proteobacteria.Alphaproteobacteria.Caulobacterales.Caulobacteraceae
Bacteria.Proteobacteria.Betaproteobacteria.Burkholderiales
Associated Group
Intestine
Hepatopancreas
V3V4.HE2 V3V4.HE3 V3V4.HE5 V3V4.HE6 V3V4.IE2 V3V4.IE3 V3V4.IE5 V3V4.IE6
1.0 1.5 2.0 2.5 3.0 3.5
LDA (Log10)
Differentially abundant features

**Figure S8.** Distribution of differentially abundant multi-level taxa selected by the LDA Effect Size (LEfSe) associated to split region and organ groups. Each sample subset (organ and region group) of the species table of the core gg97 BioSet table was analyzed independently of the rest of the samples. The heatmap represents the scaled distribution of each taxa across the samples, that is differentially associated to a group by their LEfSe. Shown taxa are preferentially detected in the indicated region-organ group. Color intensity (warmth) represents relative abundances (dark blue = 0 to light yellow = 1). LDA is on a log10 scale and features with an effect size >2 are shown. Only the highest taxonomic levels bearing any informative taxonomic labels are shown (from phylum to the highest level available for each taxon using the greengenes' nomenclature). (**a**) Differentially abundant taxa associated to each region when only hepatopancreas samples are considered. (**b**) Differentially abundant taxa associated to each region when only intestine samples are considered. (**c**) Differentially abundant taxa associated to each organ when only V3 samples are considered. (**d**) Differentially abundant taxa associated to each organ when only V4 samples are considered. (**e**) Differentially abundant taxa associated to each organ when only V3V4 samples are considered.
